# Using Regulatory and Epistatic Networks to Extend the Findings of a Genome Scan: Identifying the Gene Drivers of Pigmentation in Merino Sheep

**DOI:** 10.1371/journal.pone.0021158

**Published:** 2011-06-20

**Authors:** Elsa García-Gámez, Antonio Reverter, Vicki Whan, Sean M. McWilliam, Juan José Arranz, International Sheep Genomics Consortium, James Kijas

**Affiliations:** 1 Livestock Industries, Commonwealth Scientific and Industrial Research Organisation (CSIRO), Brisbane, Queensland, Australia; 2 Departamento de Producción Animal, Universidad de León, León, Spain; South Texas Veterans Health Care System, United States of America

## Abstract

Extending genome wide association analysis by the inclusion of gene expression data may assist in the dissection of complex traits. We examined piebald, a pigmentation phenotype in both human and Merino sheep, by analysing multiple data types using a systems approach. First, a case control analysis of 49,034 *ovine* SNP was performed which confirmed a multigenic basis for the condition. We combined these results with gene expression data from five tissue types analysed with a skin-specific microarray. Promoter sequence analysis of differentially expressed genes allowed us to reverse-engineer a regulatory network. Likewise, by testing two-loci models derived from all pair-wise comparisons across piebald-associated SNP, we generated an epistatic network. At the intersection of both networks, we identified thirteen genes with insulin-like growth factor binding protein 7 (*IGFBP7*), platelet-derived growth factor alpha (*PDGFRA*) and the tetraspanin platelet activator *CD9* at the kernel of the intersection. Further, we report a number of differentially expressed genes in regions containing highly associated SNP including *ATRN*, *DOCK7*, *FGFR1OP*, *GLI3*, *SILV* and *TBX15*. The application of network theory facilitated co-analysis of genetic variation with gene expression, recapitulated aspects of the known molecular biology of skin pigmentation and provided insights into the transcription regulation and epistatic interactions involved in piebald Merino sheep.

## Introduction

The population history and genetic structure of domestic animals offer advantages for the identification of the genetic drivers associated with phenotypic change. High throughput genotyping has lead to genome wide association studies (GWAS) which have successfully identified the genetic basis of monogenic disease in cattle [1], sheep [2] and dog [3]. Further, recent studies have identified complex traits such as skeletal morphology are under the control of a small number of genes of large effect [4]. For human and livestock populations however, the majority of traits are likely to be controlled by a larger number of genes which individually confer small effects [5], [6]. Given that most tests for association proceed in a simple SNP-by-trait fashion using stringent significance levels to offset for multiple testing, the result is many of the genes that contribute to trait variation remain undetected.

To explore approaches which seek to incorporate GWAS into systems biology, we have merged SNP variation with analysis of differential gene expression to investigate the basis of a phenotypic trait in sheep. This was prompted by recent studies which exploit gene network theory and systems approaches to identify key genetic drivers [7]–[10]. In our case, we co-analysed variation in allele frequency, gene expression and transcription factor mediated gene regulation to incriminate genes which would otherwise have remained undetected using a single data type in isolation. To test our approach we selected piebald, which in humans is a leukoderma arising from disregulation of melanocyte development and migration often caused by mutations in the *KIT* gene [11]. In sheep however, piebald, is an economically important phenotype in Australian Merino sheep characterised by the presence of one or more asymmetric pigmented regions. Test matings indicate the condition is not consistent with a simple Mendelian mode of inheritance [12] and the location and extent of pigmentation in effected animals varies considerably, suggesting the coordinated action of multiple genes.

## Results

### SNP Association and Gene Expression Confirm Piebald Has a Multigenic Basis

**Figure 1:**
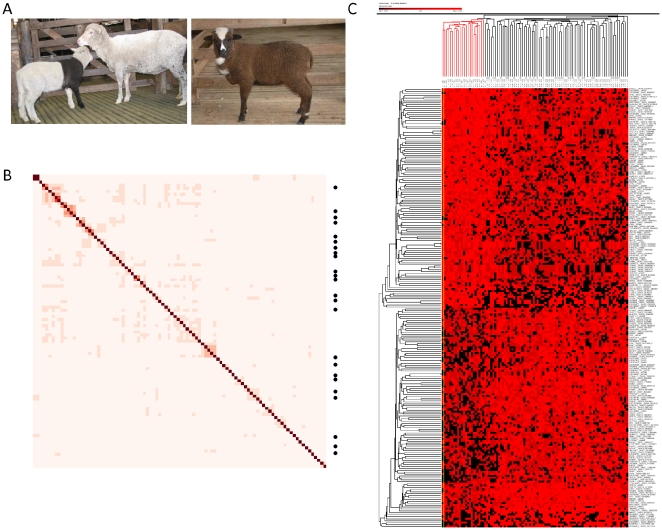
Genome wide association for piebald. (**A**) A piebald lamb is pictured with its non-pigmented mother (left hand side). The asymmetrical presentation of pigmentation (the lamb has one pigmented and one white forelimb) characterises this colour morph from *Recessive Black* (right hand side) which arises through action of the *Agouti* locus [39]. (**B**) The genetic relationship between 24 piebald animals and 72 non-pigmented controls. Allele sharing, or genetic similarity, was calculated between each pair-wise combination of animal using 48,686 SNP. Increasing values of allele sharing are represented using darker colour. Unsupervised hierarchical clustering distributed the piebald cases (indicated with a dot) across the matrix, reflecting the sampling strategy used to select controls which were as closely related to cases as possible. (**C**) Unsupervised hierarchical clustering of the 226 associated SNP (*P*<0.001) successfully distinguished all piebald animals as genetically distinct from the controls. Animals are arranged into columns and annotated above the matrix using either red (piebald) or black lines (controls). The 226 associated SNP are arranged into rows and each cell indicates the observed genotypic outcome as follows: homozygote (bright red), heterozygote (dark red), alternate homozygote (black).

We collected DNA from 24 piebald Merinos characterised by the appearance of pigmentation spots (Figure 1A). To minimise unrelated genetic variability, we then selected 72 genetically similar but non-pigmented Merinos from a wider population sample using allele sharing calculated from 49,034 SNP. The resulting relationship matrix linking all 96 animals is shown in Figure 1B. Comparing allele frequency differences between piebald and non-pigmented animals revealed 226 loci were highly associated (*p*<0.001) and collectively distinguished piebald from non-pigmented animals (Figure 1C). The highest association (*p* = 8.45×10^−7^) was observed for SNP *s49104* located in the region containing *IGFBP7* (OAR 6 Mb 78.9), however the absence of a single and strong association peak confirmed a multigenic basis for *ovine* piebald (Figure S1).

We sought to interpret these genetic associations using gene expression obtained from five skin tissue types isolated from non-pigmented, piebald and also recessive black individuals known to be under the control of *Agouti* (Figure 1A, 2A). The five tissue types were white skin tissue from non-pigmented sheep (NOR); black skin tissue from a piebald animal (PBB); white skin tissue from a piebald animal (PBW); black skin tissue from a recessive black animal (RSB) and white skin tissue from the non-pigmented region of a recessive black animal (RSW). Seven contrasts between tissue types (named DE1–DE7, see Methods section) were examined using a microarray containing 3,685 unique skin-specific genes. Of these, 54 genes displayed differential expression (DE) in ≥4 contrasts and hierarchical cluster analysis revealed coexpression across tissue types (Figure S2). A set of 19 genes, including 11 keratin family members displayed coordinated down regulation in piebald tissue, again indicating no single gene alone appeared responsible for the trait.

**Figure 2:**
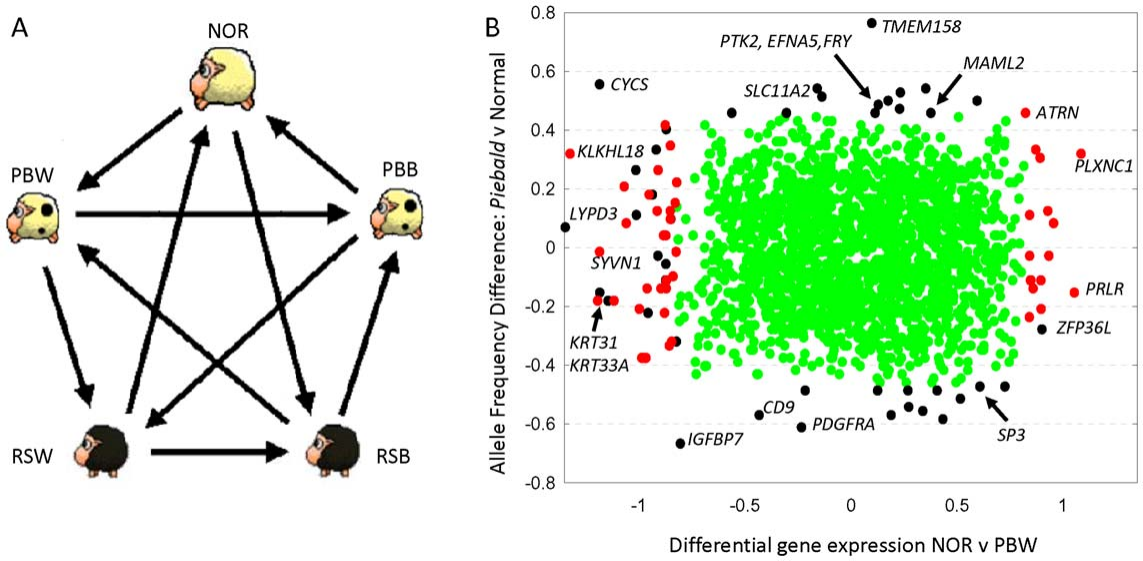
Gene expression relating to pigmentation. (**A**) Design of the microarray experiment, showing hybridizations (arrows) between five tissue types. Samples were labelled with either red (arrow head) or green dye (arrow tail) and the experiment was repeated in a dye swap using samples from independent biological replicates. A total of 20 hybridizations were performed to compare the following tissue types: white skin tissue from 4 pooled non-pigmented animals (NOR); black skin tissue from a piebald animal (PBB); white skin tissue from a piebald animal (PBW); black skin tissue from a recessive black animal (RSB) and white skin tissue sampled from the inguinal, non-pigmented area from a recessive black animal (RSW). Each sample intervened in four hybridizations (two labelled red and two labelled green). (**B**) Plot of SNP allele frequency difference between piebald and normal animals (Y-axis) and differential gene expression in contrast 3 (NOR versus PBW) (X-axis) for a set of 1,935 genes. Red symbols represent genes which were both differentially expressed (piebald versus normal) and have a SNP within 2.5 Kb. Black symbols represent genes which either displayed differential expression (*P*<0.01) or are located within 1 Mb of an associated SNP. The remaining green symbols represent genes which were neither differentially expressed nor located near associated SNP.

Charting the proximity of SNP to the genomic location of genes revealed 17,223 SNP (35%) were either intragenic or within 2.5 Kb of a gene (Figure S3). Further, 1,935 genes present on the skin-specific microarray had a genotyped SNP within 1 Mb. This allowed us to search for genes displaying both DE and genetic association relating to piebald (Figure 2B). Analysis across all seven contrasts identified a total of 370 DE genes located within 1 Mb of a SNP (Figure S4). Of these, 287 had a SNP sufficiently close (<2.5 Kb) to be considered putatively *cis*-acting. On this basis, we identified 312 ‘piebald-associated genes’ which displayed either (i) DE in one or more contrasts and a *cis*-SNP or (ii) the absence of DE but an associated SNP within the chromosomal region (<1 Mb) (Table S1). *IGFBP7* was both highly associated with piebald and down-regulated in piebald tissue (Table 1). Similarly, *ATRN*'s expression was up-regulated in piebald tissue and a putatively *cis*-acting SNP displayed strong association while *TMEM158*, *PDGFRA* and *PTK2* were not DE in any contrast, however co-localised SNP were highly associated (Table 1).

**Table 1:** A selection of key genes involved in piebald.

GeneID	Chr	Mb	Normalised Gene Expression^1^					SNP^2^	Allele Frequency^3^		P-value^4^	OR^4^
			NOR	PBB	PBW	RSB	RSW		Piebald	Non_Piebald		
*ATRN*	13	55.4	6.15	8.43	8.42	7.59	8.99	OAR13_55578882	0.67	0.44	5.96E-03	2.57
*C1QL1*	11	46.9	NA	NA	NA	NA	NA	s27884	0.19	0.5	1.47E-04	4.33
***CD9***	3	225.7	9.63	9.32	9.01	8.33	9.58	s65742	0.29	0.58	6.34E-04	3.3
*DOCK7*	1	38.6	2.47	4.18	4.36	4.67	4.18	OAR1_38581202	0.67	0.74	3.05E-01	1.45
***EFNA5***	5	112.9	8.44	9.15	8.91	8.59	9.15	s72651	0.58	0.79	4.40E-03	2.71
*FGFR1OP*	8	95.4	7.87	6.73	7.28	6.75	7.09	OAR8_95443730	0.9	0.81	1.80E-01	1.98
***FRY***	10	29.2	6.78	8.5	8.19	7.97	8.81	OAR10_29223007	0.69	0.42	1.54E-03	2.99
*GLI3*	4	84	5.85	7.48	7.58	6.81	8.39	OAR4_83976143	0.67	0.78	9.95E-02	1.82
***IGFBP7***	6	78.9	11.37	10.35	9.7	9.87	10.7	s49104	0.54	0.88	8.45E-07	5.92
*KRT31*	11	43.8	14.06	11.25	12.11	12.77	12.57	OAR11_43737226	0.71	0.67	6.55E-01	1.18
***LARP7***	6	15.7	6.29	7.99	7.71	6.51	8.76	s14758	0.46	0.67	7.80E-03	2.44
***MAML2***	15	13.4	7.05	7.57	8.31	7.34	8.6	s71238	0.88	0.65	2.62E-03	3.84
***MYH10***	11	29.3	5.36	5.77	5.97	6.43	7.54	s29973	0.46	0.74	2.74E-04	3.42
***PDGFRA***	6	76.2	6.12	5.33	6.22	5.99	6.1	OAR6_76377079	0.31	0.62	2.34E-04	3.56
***PTK2***	9	16.2	5.06	6.12	5.75	4.78	5.73	s22485	0.58	0.35	5.20E-03	2.55
***PTPN18***	2	121.1	6.99	7.19	7.87	7.54	8.69	s23889	0.71	0.46	2.68E-03	2.87
*SHISA9*	24	13.2	7.23	7.57	8.31	8.38	8.88	OAR24_13240468	0.9	0.63	4.28E-04	5.16
*SILV*	3	174.6	8.16	8.87	8.51	9.15	9.22	s59363	0.79	0.87	2.01E-01	1.73
***SLC11A2***	3	144.3	7.76	7.44	7.52	7.89	8.05	OAR3_144283427	0.88	0.64	2.03E-03	3.96
***SP3***	2	143.5	6.2	7.36	8.33	7.49	8.29	OAR2_143746835	0.54	0.78	1.63E-03	2.96
*TBX15*	1	101.9	4.38	3.67	4.96	5.29	4.53	OAR1_101890858	0.54	0.6	4.46E-01	1.29
***TMEM158***	19	57.1	6.18	6.95	6.76	6.41	7.77	s04445	0.79	0.41	4.57E-06	5.47

A selection of key genes differentially expressed (DE; *P*<0.01) in at least one of the seven contrasts and/or near a SNP associated with the piebald conditions (*P*<0.001).

1 The mean normalised gene expression is given for each of five tissue types (NOR, PBB, PBW, RSB and RSW as define in Figure 2A). Values for piebald tissue (PBB or PBW) that are higher than in the normal tissue (NOR) indicate up regulation (eg *ATRN*).

2 SNP with the highest association within a 1 Mb region centered on the gene.

3 Allele frequencies are given in both the piebald and non-piebald animal populations.

**Figure 3:**
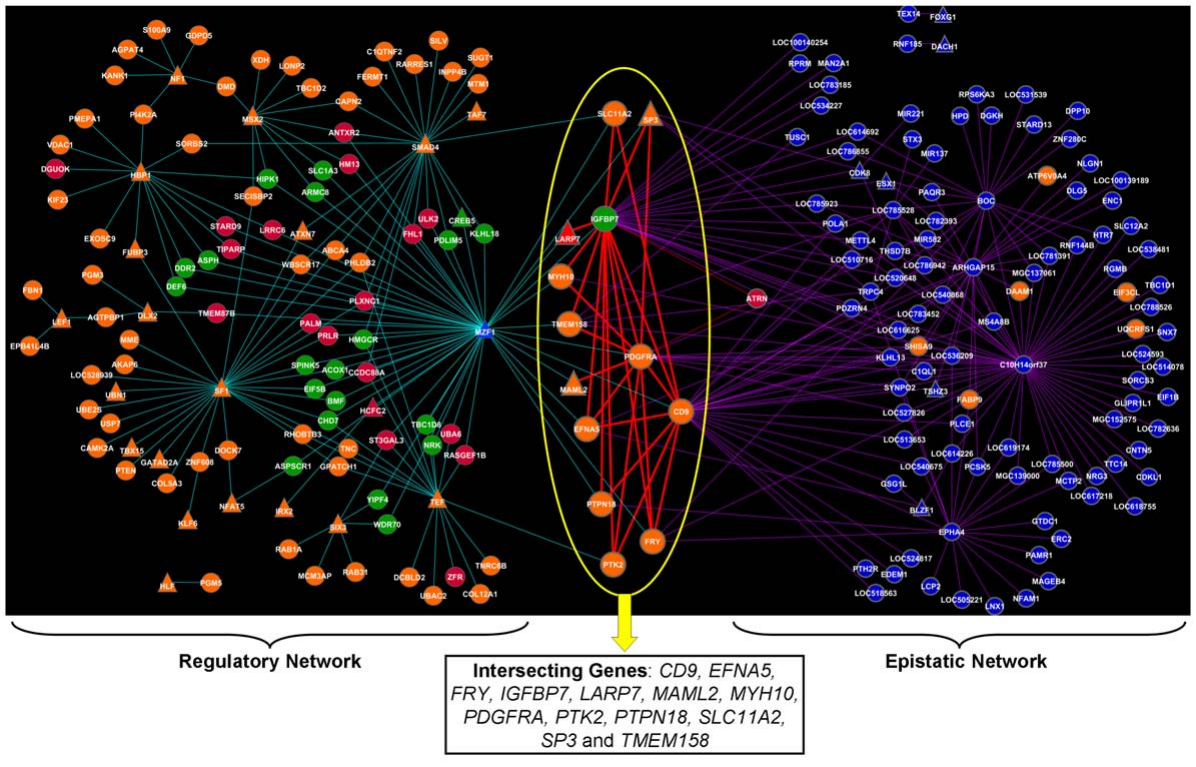
Regulatory and epistatic networks. Promoter sequence analysis of piebald-associated genes (Table S1) was performed to identify transcription factors (TF; represented by triangles) and build a regulatory network (left panel). Two-loci models for all pairwise comparisons among piebald-associated SNPs were fitted to develop an epistatic network (right panel). The overall landscape revealed the presence of thirteen genes located at the intersection of both networks: *CD9*, *EFNA5*, *FRY*, *IGFBP7*, *LARP7*, *MAML2*, *MYH10*, *PDGFRA*, *PTK2*, *PTPN18*, *SLC11A2*, *SP3* and *TMEM158*. Gene color indicates over-expression (red), under-expression (green), unchanged expression (orange) and genes not represented on the microarray (blue) in highly correlated contrasts (DE3, DE5 and DE6). The network file (*.cys) is available on request from the authors to explore within Cytoscape [46].

4 Both the *P*-value and associated odd ratio (OR) are given for SNP^2^. See Table S1 for full list of 312 key genes. Genes in bold type correspond to genes located at the intersection between the regulatory and the epistatic network (Figure 3).

### Regulatory and Epistatic Networks Identify the Gene Drivers of Pigmentation

A network was constructed to explore regulation of DE genes through the action of transcription factors (TF). First, promoter sequence analysis of each piebald-associated gene was performed to identify the complement of transcription factor binding sites (TFBS) associated with each. Then, a regulatory network was constructed where nodes represent genes. The presence of a TFBS created an edge linking a gene with the TF for which it contained a binding site. Gene expression was also used as input using the highest correlated contrasts (DE3, DE5 and DE6; Figure S5). The network was visualised using the Cytoscape software, as described in the methods. Fourteen TFs were present in the network (*MZF1*, *SF1*, *SMAD4*, *TEF*, *HBP1*, *MSX2*, *NF1*, *SIX3*, *DLX2*, *LEF1*, *NFAT5*, *HLF*, *IRX2* and *KLF6*), none of which displayed DE, but collectively linked 108 piebald-associated genes. Of these, three (*MZF1*, *SF1* and *SMAD4*) have a binding site in 20 or more piebald-associated genes (Figure 3), strongly suggests a pivotal role in regulation. Importantly, *SMAD4* inhibits *PAX3* [13] which is a key regulator of *MITF* that regulates the degree of black spotting in both dog [14] and cattle [6]. In our GWAS, SNP *OAR19_33605872* (located 310 Kb from *MITF*) shows suggestive association to piebald (*P* = 7.05E-03).

A second network was built to explore epistatic interactions between SNP and their closest genes. In this approach, all pair-wise combinations (25,425 pairs) of the 226 SNP found to be associated with piebald (Figure 1C) were tested for evidence of epistasis. A total of 645 significant pairs were identified (*p*<0.001) which served as input data for the epistatic network. To reduce network complexity, ‘hubs’ present in more than 20 significant pairs were identified (*ARHGAP15*, *BOC*, *C10H14orf37*, *CD9*, *EPHA4*, *IGFBP7*, *LOC785528* and *TMEM158*) and used to extract edges in a reduced network containing 121 nodes (genes) and 216 edges (Figure 3). This revealed *C10H14orf37* as the hub with the highest number of connections (61 different pairs). *IGFBP7* was the next most connected (32 pairs), ten of which were also significant for *PDGFRA*. Moreover, as a pair, *IGFBP7* and *PDGFRA* were able to distinguish between piebald and non-pigmented animals (*P* = 0.000053). Interestingly, *MAML2* and *PDGFRA* paired together suggesting a regulatory interaction however this could not be evaluated as *MAML2* is a non-DNA binding transcription co-activator.

The final analysis identified thirteen genes that were common to both the regulatory and epistatic networks. These formed the intersecting landscape displayed in Figure 3 (*CD9*, *EFNA5*, *FRY*, *IGFPB7*, *LARP7*, *MAML2*, *MHY10*, *PDGFRA*, *PTK2*, *PTPN18*, *SLC11A2*, *SP3* and *TMEM58*). Three transcription factors created all of the links from the regulatory network into the intersecting genes (*MZF1*, *TEF* and *SMAD4*) while an additional two transcription factors were present within the intersecting landscape via their connection to other genes made by epistatic interactions (*MAML2* and *SP3*).

## Discussion

The fundamental processes behind melanocyte differentiation and migration are well understood thanks largely to their relevance in the context of human melanoma and vitiligo [11]. Many cases involve disruption of a single gene, however analysis in this study failed to identify a gene of large effect via SNP based association testing. Therefore, the approach developed sought to investigate the genetic control of phenotypic variation through co-analysis of divergent data types. In our case, the three data types were genomic variation (SNP) which differed between case and control populations, gene expression differences derived from symptomatic versus asymptomatic tissues and transcription factor mediated gene regulation. We searched the resulting data for the coordinated regulation of genes via transcription factors (Figure 3, left side) and we reasoned that the coordinated action of multiple genes could be detected through analysis for epistatic interactions at the genetic level (Figure 3, right side). Using network theory, we then interrogated the results of both approaches and identified a small number of intersecting genes. This clearly revealed the molecular complexity underpinning the piebald phenotype in Merino sheep.

The current study has two technical limitations likely to have restricted our power to detect genes which contribute to piebald. Firstly, the microarray used to detect gene expression contained only a subset of sheep transcripts. This may not have been a critical limitation when investigating a pigmentation trait given the microarray was intentionally populated with 6,125 skin expressed sequence tags [15], however the possibility remains that biologically important genes were unavailable for testing. Secondly, the *ovine* SNP chip facilitated genotyping of 49,034 SNP however only 35% of these are located with 2.5 Kb of a gene. This meant many genes were not tagged by a physically associated genetic marker and recombination (between SNP and their nearest gene) is likely to have eroded the strength of association in many cases. In addition to these technical considerations, it is worthwhile noting the analytical approach likely influenced the genes identified. Network theory has long recognised the powerful role that hubs play in networks, namely their ability to pervade most of the network topology and provide robustness [16]. We reduced network complexity by identification of hubs, but acknowledge this may have resulted in biologically relevant genes being missed where they do not act as hubs.

Despite these considerations, our approach has identified genes which would have remained hidden using either data type in isolation. In what follows, and as a proof of concept validating the biological relevance of our results, we describe how genes located within in the intersecting landscape of the joint network (Figure 3) are known to be involved in pigmentation and more specifically in vitiligo. CD9 is a cell surface protein member of the tetraspanin family mediating signal transduction events that play a role in the regulation of cell development, activation, growth and motility [17] including motility of epidermal keratinocytes [18], [19]. The finding that it can trigger platelet activation [20] makes our finding of an epistatic SNP pair connecting *CD9* and *PDGFRA* highly relevant. Indeed, one of our main candidates underpinning the piebald phenotype is *PDGFRA* taking, along with *CD9* and *IGFBP7*, a prominent hub role in the landscape intersecting the regulatory and the epistatic networks. Xu et al. [21] speculate that the *PDGFRA* gene may be a candidate susceptibility gene of vitiligo. Furthermore, within the context of livestock species a genome-wide scan [22] found the gene *PDGFRA* to be under positive selection in Montbéliarde breed of cattle possibly underlying the primary importance of coat color patterns for herd-book registration. Another intersecting genes (Figure 3) is insulin-like growth factor-binding protein 7 (IGFBP7) which is a secreted protein and functions extracellularly. Hochberg et al. [23] first showed that IGFBP7 is underexpressed in psoriatic epidermis but is inducible by ultraviolet treatment. Since then, IGFBP7 has received particular attention due to its potential to induce apoptosis in human melanoma cell lines [24], [25]. Of particular relevance is the fact that both *PDGFRA* and *IGFBP7* are located within a 3 Mb window of ovine chromosome 6 (OAR6), in a region that also contains the *KIT* gene to which piebald was first associated [26]. In this pioneering work, the authors did not exclude the involvement of other closely linked genes such as *PDGFRA*. In order to further shed light into this issue, we took a detailed look at the LD structure and Manhattan plot of the SNP association results on OAR6 from 75 to 80 Mb (Figure S6). The SNP associated to piebald (SNP ID: OAR6_76377079; P<1.0E-3) is 116.0 kb downstream from *PDGFRA* and 277.7 kb upstream from *KIT*. Hence, the association of *KIT* with piebald in sheep cannot be entirely disregarded.

Two additional genes found within the intersection between the regulatory and the epistatic networks are *PTK2* and *EFNA5*. Protein tyrosine kinase 2 (*PTK2*), also known as *FAK*, has been reported to stimulate melanocyte migration [27] and to induce vitiligo repigmentation in vitro [28]. *EFNA5* is a member of the ephrin family of proteins, components of cell signalling pathways involved in development [29]. Its role as a negative regulator of epidermal growth factor receptor (*EGFR*) has recently been established [30]. Equally relevant, mutations in *EGFR* have been reported to be associated with dark skin in mice [31], however the gene was not significantly associated in this study. We did find, however, that expression of *HBEGF* was 1.91-fold down regulated (*P* = 8.14E-3) in white compared to black samples (DE1 and DE7; Table S1) and a SNP in its coding region (*OAR5_53183086*) also displayed suggestive association (*P* = 0.0894).

Finally, we surveyed the Color Genes database [32] and found it to include a number of genes reported in our study as either being DE and/or harbouring a SNP associated with the piebald phenotype. These include: *ATRN*, *DOCK7*, *FGFR1OP*, *GLI3*, *SILV* and *TBX15* (Table 1). The molecular role of *ATRN* as a crosstalk between melanocortin-receptor signalling and immune function was first reported by [33]. Further, He et al. [34] established that *ATRN* is a transmembrane receptor for agouti protein. More recently, Seo et al. [35] reviewed the biology of epidermal and hair pigmentation in cattle and highlighted the likely role of *ATRN* in he switching between the synthesis of melanin components. A similar relationship with the agouti protein exists with TBX15, DOCK7 and GLI3. In the absence of TBX15, expression of agouti in mice is displaced dorsally [36]. Mice with mutations in the dedicator of cytokinesis 7 protein (DOCK7) have generalized hypopigmentation and white-spotting [37]. Matera et al. [38] showed that loss of GLI3 signalling disrupts melanoblast specification and that a mutation in *GLI3* causes increased hypopigmentation in mice.

In conclusion, the incorporation of experiments which utilise multiple data types in a systems biology context is an appropriate approach for understanding complex biological systems. Whole genome sequencing will soon become viable for livestock [39]. This will bring access to every SNP and structural variant differing between the genomes of case and control populations. When combined with methods to measure global transcription from target tissues, the approach described here holds enormous promise for the elucidation of the genetic drivers which underpin a spectrum of traits which, to this point, have remained intractable.

## Materials and Methods

### Piebald Animal Resource, Genotyping and Testing for Association

Pigmented Merinos within Australian industry flocks had the location and extent of colour spots photographically recorded. Care was taken to avoid inclusion of cases which displayed symmetrical pigmentation which is diagnostic of the action of *Agouti* in sheep [40]. Piebald animals (n = 24) from 23 properties were selected for genotyping to reduce relatedness between cases. Genomic DNA was genotyped using the *Ovine* SNP50 BeadChip (http://www.illumina.com) before raw signal intensities were converted into genotype calls using Illumina's Genome Studio software. SNP were pruned using a series of quality filters to define a final set of 49,034 SNP. Breed matched control animals (non piebald) were selected from the 199 Australian Merinos genotyped as part of the International Sheep Genomics Consortium's HapMap project (www.sheephapmap.org). Pair-wise allele sharing was calculated between all animals (24 cases and 199 controls) from all SNP. The resulting allele sharing matrix was used in a step wise fashion to select 72 control animals which maximised allele sharing between cases and controls, thereby reducing genetic variability and substructure unrelated to piebald. The filtering schema proceeded as follows: for each piebald individual, three non-pigmented controls with the highest pair-wise allele sharing were selected. Once a normal individual was selected, it was not included in the search of the three most related individuals with the next piebald individual. We measured the statistical association of each of SNP with piebald as the difference between its average genotype across within the piebald sample (24 animals) minus its average genotype across the normal sheep (72 animals). SNPs were deemed to be significantly associated if the observed genotype difference was beyond three standard deviations from the mean and the chi-square test yielded a P-value<0.001.

### Linking Gene Expression to GWAS

SNP was mapped with its nearest protein-coding gene using the available sheep genome sequence [39]. The final data set included 48,686 SNP that displayed polymorphism within the 96 cases and controls. Of these, 15,624 SNPs were intragenic, while a further 1,592 SNPs were located within 2.5 kb for either side of a gene's coding sequence and considered to be putatively *cis*-acting. A further 24,727 SNP were mapped beyond 20 kb of a gene and considered to be linked to another gene [41]. Figure S3 shows the empirical density distribution of the 6,687 SNPs located from 1 base-pair to 20 kb distance from a gene. Finally, 1,741 SNPs were unmapped.

### Study Design and Tissue Sampling for Gene Expression

We used the bovine/ovine skin gene expression microarray platform described previously [15]. In brief, 11,689 probes were printed in duplicate onto Corning UltraGAPS (Corning Inc., NY, USA) glass slides at a spacing of 210 µm. For skin biopsies, a wool staple sample was removed by close clipping a region of approximately 5 cm^2^. Two skin trephines of 0.9 cm diameter were taken from the clipped region and immediately placed in RNA later (Ambion) for subsequent RNA extraction. Skin samples in RNA later were placed into −80°C freezers for long-term storage. Total RNA was prepared from the skin samples using TRI Reagent in accordance with the manufacturer's recommendations (Sigma, St Louis, MO, USA).

The general experimental design for the microarray hybridisations is shown in Figure 2A. In total, 20 hybridizations were performed. The design took into account the limited animal material and was developed with an emphasis on the exploration of all possible contrasts of interest so that all samples were compared against each other in a series of hybridizations with alternate dye-swaps. Five skin samples, or experimental conditions, were explored and codified as follows:

NOR (‘normal’) = White sample from normal sheep;
PBW (‘piebald white’) = White sample from piebald sheep;
PBB (‘piebald black’) = Black sample from piebald sheep;
RSW (‘recessive white’) = White sample from *Agouti* recessive black sheep (sample taken from the inguinal, non-pigmented area); and
RSB (‘recessive black’) = Black sample from *Agouti* recessive black sheep.

We used the GenePix 4000A optical scanner and the GenePixPro 5.1 image analysis software (both from Molecular Devices, Sunnyvale, CA, USA) to quantify the gene expression level intensities. Two filtering criteria were applied during data acquisition. Firstly, probes with a signal to noise ratio less than two in all hybridisations were deemed undetectable and removed from the analysis. Secondly, for genes represented by multiple probes the most abundant probe, averaged across all hybridisations, was used. This second criterion is based on the fact that abundant probes are better annotated and their intensity signals less prone to noise. After filtering, 300,480 gene expression intensity readings remained (half from each colour channel and 15,024 from each chip) from 3,685 unique skin-specific genes. Prior to normalization, signals were background corrected and base-2 log-transformed. The arithmetic mean and standard deviation (in brackets) for the red and green intensities were 7.04 (3.24) and 8.49 (2.19) respectively. The expression data from the entire set of 20 hybridisations was deposited in Gene Expression Omnibus (GEO; http://www.ncbi.nlm.nih.gov/geo/) and can be downloaded and can be accessed using accession number GSE24189.

### Normalization of Gene Expression

Following previously described approaches [42] we fitted the following ANOVA mixed-effect model to normalize the gene expression data:
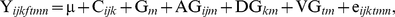
where Y*_ijkftmn_* represents the *n*-th background-adjusted, base-2 log-intensity from the *m*-th gene at the *t*-th sample variety (NOR, PBW, PBB, RSW, and RSB) taken from the *i*-th array, *j*-th printing block and *k*-th dye channel; μ is the overall mean; C represents a comparison group fixed effect defined as those intensity measurements that originate from the same array slide, printing block and dye channel; G represents the random gene effects with 3,685 levels; AG, DG, and VG are the random interaction effects of array×gene, dye×gene, and variety×gene, respectively; and e is the random error term. Using standard stochastic assumptions, the effects of G, AG, DG, VG and e were assumed to follow a normal distribution with zero mean and between-gene, between-gene within-array, between-gene within-dye, between-gene within-variety and within-gene components of variance, respectively. Restricted maximum likelihood estimates of variance components and solutions to model effects were obtained using VCE6 software (ftp://ftp.tzv.fal.de/pub/vce6/). The solutions to the VG effect were used as the normalized mean expression of each gene in each of the five samples under scrutiny.

To contrast the expression of each gene across sample types, we explored the following seven measures of differential expression (DE):

DE1 (‘black vs white within piebald’) = PBB−PBW
DE2 (‘piebald vs recessive within black’) = PBB−RSB
DE3 (‘piebald vs normal within white’) = PBW−NOR
DE4 (‘piebald vs recessive within white’) = PBW−RSW
DE5 (‘piebald vs others within white’) = PBW−½(NOR+RSW)
DE6 (‘piebald vs non-piebald’) = ½(PBW+PBB)−1/3(NOR+RSW+RSB)
DE7 (‘black vs white’) = ½(PBB+RSB)−1/3(NOR+PBW+RSW)

Using a nominal P-value<0.01 from a two-tailed t-test statistic, genes were deemed to be DE if their normalized measure of differential expression across any of the 7 DE contrasts felt beyond 2.57 standard deviations. We used PermutMatrix [43] to perform hierarchical cluster analysis of gene expression across skin tissue samples.

### Promoter sequence analysis

The bovine genome-wide promoter sequence database from Genomatix (http://www.genomatix.de/; ElDorado Btau 4, v-07-09) was used in the absence of annotated promoter data for sheep. A total of 60,131 promoter sequences derived from 22,050 genes were downloaded. To ensure only high confidence promoters were selected we applied the concept of orthologous promoters [44] and retained only those promoters for which phylogenetically conserved sequences were documented in both the human and mouse genomes. This resulted in identification of 39,696 promoter sequences distributed over 13,623 genes. We subsequently applied a threshold of 1 (100% confidence) to core and matrix similarities [45] to identify a final set of 310,316 high confidence TFBS that were used for integration with the gene expression data.

### Regulatory and Epistatic Networks

The gene regulatory network (Figure 3, left side) was constructed using two attribute types. First, output from the promoter sequence analysis was used to create edges linking genes and transcription factors (TFs). Specifically, edges were constructed only where analysis revealed a given gene contained a transcription factor binding site (TFBS) corresponding to the linked TF. Second, differential gene expression observed between piebald and non-piebald tissue was used. To obtain a global understanding of gene expression across the seven tissue contrasts (DE1–7), we first calculated the correlation (R-value) for each pair-wise combination (all 21 combinations are plotted in Figure S5). This revealed the highest three correlations (R = 0.88, 0.82 and 0.79) were observed for combinations (DE3, DE5 and DE6) which in each case compared a piebald (only white or all piebald) versus a non-piebald sample (using the normal sample, all the white non-piebald or all the non-piebald, respectively). For each gene, we therefore used the average value (from DE3, DE5 and DE6) as input in the network to show genes as either over-expressed (red), under-expressed (green) or having unchanged expression (orange) in the piebald condition. Using the two attributes the network was joined, visualised and explored using the Cytoscape software [46]. For the epistatic network (Figure 3, right side) SNP pairs were used as input which displayed evidence for epistasis. Contingency tables comprising 9 rows (combinations of two loci genotypes) and 2 columns (containing genotype counts within either the piebald or non-piebald populations) were constructed for each of the 25,425 pair-wise combinations of 226 SNP significantly associated with the piebald condition. The distribution of observations in each table were tested (P<0.001 from a chi-square test with 8 degrees of freedom) to identify 645 significant pairs. A subset of these significant pairs may result from linkage disequilibrium (as opposed to epistasis) where SNP pairs are in close physical proximity (<100 Kb). To generate the epistatic network using this data, we used the closest gene to each SNP in a significant pair. As for the regulatory network, DE was incorporated from the average of DE3, DE5 and DE6 and Cytoscape was used to built and explore the network [46].

## Supporting Information

Figure S1 Genome wide association results for piebald. The strength of association expressed as negative log *P* values (Y axis) are shown for 49034 SNP (X axis) arranged in genomic order from chromosome 1 (far left) to the X chromosome (far right).
(TIF)

Figure S2 Co-regulation of 54 genes associated with pigmentation. Gene expression was examined using five tissue types as follows: white skin tissue from a non-pigmented animal (NOR); black skin tissue from a piebald animal (PBB); white skin tissue from a piebald animal (PBW); black skin tissue from a self color black animal (RSB) and white skin tissue from a self color black animal (RSW). The normalised mean expression (NME) within each tissue type is represented using color ranging from green (down regulation) through to red (up regulation). The 54 genes displayed were differentially expressed in at least 4 of the 7 tissue type contrasts examined (Figure 1 describes the 7 contrasts). Hierarchical clustering was performed to identify genes which are expressed in a coordinated way across tissue types, which revealed a set of 11 keratin genes strongly down regulated in piebald tissue.
(TIF)

Figure S3 Physical proximity of SNP on the ovine SNP50 BeadChip to genes annotated in Ovine Genome Assembly v1.0 (https://www.biolives.csiro.au/cgi-bin/gbrowse/oar1.0/). A total of 47,275 SNP with known base pair location were examined. Of these, 15,624 SNP (or 33%) were intragenic while 24,921 (53%) were located greater than 20 Kb from the nearest gene. The distribution of the remaining 6,730 (14%) of SNP is shown where the SNP to gene distance was binned in increments of 0.2 Kb from 1 bp to 20 Kb. Importantly, the analysis reported 17,223 SNP were located with 2.5 Kb of the nearest gene which was our empirical threshold for defining SNP as potentially be *cis*-acting.
(TIF)

Figure S4 Plot of allele frequency difference versus gene expression for a set of 1,935 genes. For each of seven gene expression contrasts (termed DE1–DE7; refer to the materials and methods), the position of each symbol plots both the SNP allele frequency difference between piebald and non-piebald animals (Y axis) and differential gene expression (X axis). Red symbols represent genes which were both differentially expressed (piebald versus normal) and have a SNP within 2.5 Kb. Black symbols represent genes which either (i) displayed differential expression (p-value<0.05) or (ii) are located within 1 Mb of an associated SNP (p-value<0.01). The remaining green symbols represent genes which were neither differentially expressed nor located near associated SNP.
(TIF)

Figure S5 Correlation of differential expression for 1,935 genes. Differential gene expression observed within each of seven contrasts (termed DE1–DE7) were correlated in 21 pair-wise comparisons. The highest correlation (R = 0.88) was observed between DE3 and DE5, both of which examined the difference between piebald and non-piebald tissues. The only difference between the two contrasts being inclusion of an additional tissue type (RSW) in DE5. Similarly, the second and third highest correlations (DE5 and DE6 R = 0.82; DE3 and DE6 R = 0.79) was also found between contrasts constructed between piebald and non-piebald tissue types. Three contrasts together (DE3, DE5 and DE6) were used to assign genes as either over-expressed (red), under-expressed (green) or having unchanged expression (orange) in the gene networks relating to piebald (Figure 3).
(TIF)

Figure S6 SNP association results for OAR6. (**A**) The strength of association between each SNP and piebald is given as negative log P values (X axis) across a 5 Mb region of sheep chromosome (OAR) 6 (X axis). This shows the position of the highest ranked SNP (*s49104*), genome wide, at Mb position 79.9. (**B**) Pair-wise linkage disequilibrium between SNP, measured as *r* ^2^, was calculated using all 96 animals (24 cases and 72 controls) and plotted as a heatmap in Haploview. This shows LD extends for only short distances across the region. The relative location of each annotated gene within the region is shown in (**C**).
(TIF)

Table S1 Piebald associated genes. All of the genes listed displayed either DE in one or more contrasts and a *cis*-SNP or the absence of DE but an associated SNP within the chromosomal region (<1 Mb).
(DOC)

## References

[1] Charlier C, Coppieters W, Rollin F, Desmecht D, Agerholm JS (2008). Highly effective SNP-based association mapping and management of recessive defects in livestock.. Nat Genet.

[2] Becker D, Tetens J, Brunner A, Bürstel D, Ganter M (2010). Microphthalmia in Texel sheep is associated with a missense mutation in the paired-like homeodomain 3 (PITX3) gene.. PLoS One.

[3] Parker HG, Shearin AL, Ostrander EA (2010). Man's best friend becomes biology's best in show: genome analyses in the domestic dog.. Annu Rev Genet.

[4] Boyko AR, Quignon P, Li L, Schoenebeck JJ, Degenhardt JD (2010). A simple genetic architecture underlies morphological variation in dogs.. PLoS Biol.

[5] Yang J, Benyamin B, McEvoy BP, Gordon S, Henders AK (2010). Common SNPs explain a large proportion of the heritability for human height.. Nat Genet.

[6] Hayes BJ, Pryce J, Chamberlain AJ, Bowman PJ, Goddard ME (2010). Genetic architecture of complex traits and accuracy of genomic prediction: coat colour, milk-fat percentage, and type in Holstein cattle as contrasting model traits.. PLoS Genetics.

[7] Fortes MR, Reverter A, Zhang Y, Collis E, Nagaraj SH (2010). Association weight matrix for the genetic dissection of puberty in beef cattle.. Proc Natl Acad Sci USA.

[8] Peidis P, Giannakouros T, Burow ME, Williams RW, Scott RE (2010). Systems genetics analyses predict a transcription role for P2P-R: molecular confirmation that P2P-R is a transcriptional co-repressor.. BMC Syst Biol.

[9] Diez D, Wheelock AM, Goto S, Haeggström JZ, Paulsson-Berne G (2010). The use of network analyses for elucidating mechanisms in cardiovascular disease.. Mol Biosyst.

[10] Hecker M, Lambeck S, Toepfer S, van Someren E, Guthke R (2009). Gene regulatory network inference: data integration in dynamic models-a review.. Biosystems.

[11] Bondanza S, Bellini M, Roversi G, Raskovic D, Maurelli R (2006). Piebald trait: Implication of kit mutation on in vitro melanocyte survival and on the clinical application of cultured epidermal autografts.. J Invest Dermatol.

[12] Brooker MG, Dolling CHS (1969). Pigmentation of sheep: III. Piebald pattern in the Merino.. Australian J Agricultural Research.

[13] Yang, Li Y, Nishimura EK, Xin H, Zhou A (2008). Inhibition of PAX3 by TGF-β modulates melanocyte viability.. Mol Cell.

[14] Karlsson EK, Baranowska I, Wade CM, Salmon Hillbertz NH, Zody MC (2007). mapping of mendelian traits in dogs through genome-wide association.. Nat Genet.

[15] Smith WJ, Li Y, Ingham A, Collis E, McWilliam SM (2010). A genomics-informed, SNP association study reveals FBLN1 and FABP4 as contributing to resistance to fleece rot in Australian Merino sheep.. BMC Vet Res.

[16] Barabasi A, Oltvai Z (2004). Network biology: Understanding the cell's functional organization.. Nat Rev.

[17] Charrin S, Le Naour F, Oualid M, Billard M, Faure G (2001). The major CD9 and CD81 molecular partner. Identification and characterization of the complexes.. J Biol Chem.

[18] Baudoux B, Castanares-Zapatero D, Leclercq-Smekens M, Berna N, Poumay Y (2000). The tetraspanin CD9 associates with the integrin alpha6beta4 in cultured human epidermal keratinocytes and is involved in cell motility.. Eur J Cell Biol.

[19] Peñas PF, García-Díez A, Sánchez-Madrid F, Yáñez-Mó M (2000). Tetraspanins are localized at motility-related structures and involved in normal human keratinocyte wound healing migration.. J Invest Dermatol.

[20] Lanza F, Wolf D, Fox CF, Kieffer N, Seyer JM (1991). cDNA cloning and expression of platelet p24/CD9. Evidence for a new family of multiple membrane-spanning proteins.. J Biol Chem.

[21] Xu S, Zhou Y, Yang S, Ren Y, Zhang C (2010). Platelet-derived growth factor receptor alpha gene mutations in vitiligo vulgaris.. Acta Derm Venereol.

[22] Flori L, Fritz S, Jaffrézic F, Boussaha M, Gut I (2009). The genome response to artificial selection: a case study in dairy cattle.. PLoS One.

[23] Hochberg M, Zeligson S, Amariglio N, Rechavi G, Ingber A (2007). Genomic-scale analysis of psoriatic skin reveals differentially expressed insulin-like growth factor-binding protein-7 after phototherapy.. Br J Dermatol.

[24] Wajapeyee N, Serra RW, Zhu X, Mahalingam M, Green MR (2008). Oncogenic BRAF induces senescence and apoptosis through pathways mediated by the secreted protein IGFBP7.. Cell.

[25] Scurr LL, Pupo GM, Becker TM, Lai K, Schrama D (2010). IGFBP7 is not required for B-RAF-induced melanocyte senescence.. Cell.

[26] Fleischman RA, Saltman DL, Stastny V, Zneimer S (1991). Deletion of the c-kit protooncogene in the human developmental defect piebald trait.. Proc Natl Acad Sci USA.

[27] Wu CS, Lan CC, Chiou MH, Yu HS (2006). Basic fibroblast growth factor promotes melanocyte migration via increased expression of p125(FAK) on melanocytes.. Acta Derm Venereol.

[28] Lan CC, Wu CS, Chiou MH, Hsieh PC, Yu HS (2006). Low-energy helium-neon laser induces locomotion of the immature melanoblasts and promotes melanogenesis of the more differentiated melanoblasts: recapitulation of vitiligo repigmentation in vitro.. J Invest Dermatol.

[29] Boyd AW, Lackmann M (2001). Signals from Eph and ephrin proteins: a developmental tool kit.. Sci STKE.

[30] Li JJ, Liu DP, Liu GT, Xie D (2009). EphrinA5 acts as a tumor suppressor in glioma by negative regulation of epidermal growth factor receptor.. Oncogene.

[31] Fitch KR, McGowan KA, van Raamsdonk CD, Fuchs H, Lee D (2003). Genetics of dark skin in mice.. Genes Dev.

[32] International Federation of Pigment Cell Societies Color Genes Database.. http://www.espcr.org/micemut/.

[33] Gunn TM, Miller KA, He L, Hyman RW, Davis RW (1999). The mouse mahogany locus encodes a transmembrane form of human attractin.. Nature.

[34] He L, Gunn TM, Bouley DM, Lu XY, Watson SJ (2001). A biochemical function for attractin in agouti-induced pigmentation and obesity.. Nat Genet.

[35] Seo K, Mohanty TR, Choi T, Hwang I (2007). Biology of epidermal and hair pigmentation in cattle: a mini-review.. Vet Dermatol.

[36] Candille SI, Van Raamsdonk CD, Chen C, Kuijper S, Chen-Tsai Y (2004). Dorsoventral patterning of the mouse coat by Tbx15.. PLoS Biol.

[37] Blasius AL, Brandl K, Crozat K, Xia Y, Khovananth K (2009). Mice with mutations of Dock7 have generalized hypopigmentation and white-spotting but show normal neurological function.. Proc Natl Acad Sci USA.

[38] Matera I, Watkins-Chow DE, Loftus SK, Hou L, Incao A (2008). A sensitized mutagenesis screen identifies Gli3 as a modifier of Sox10 neurocristopathy.. Hum Mol Genet.

[39] Archibald AL, Cockett NE, Dalrymple BP, Faraut T, The International Sheep Genomics Consortium (2010). The sheep genome reference sequence: a work in progress.. Anim Genet.

[40] Norris BJ, Whan VA (2008). A gene duplication affecting expression of the ovine ASIP gene is responsible for white and black sheep.. Genome Res.

[41] Robertson G, Hirst M, Bainbridge M, Bilenky M, Zhao Y (2007). Genome-wide profiles of STAT1 DNA association using chromatin immunoprecipitation and massively parallel sequencing.. Nat Methods.

[42] Reverter A, Barris W, McWilliam S, Byrne KA, Wang YH (2005). Validation of alternative methods of data normalization in gene co-expression studies.. Bioinformatics.

[43] Caraux G, Pinloche S (2005). PermutMatrix: a graphical environment to arrange gene expression profiles in optimal linear order.. Bioinformatics.

[44] Buske FA, Bodén M, Bauer DC, Bailey TL (2010). Assigning roles to DNA regulatory motifs using comparative genomics.. Bioinformatics.

[45] Cartharius K, Frech K, Grote K, Klocke B, Haltmeier M (2005). MatInspector and beyond: promoter analysis based on transcription factor binding sites.. Bioinformatics.

[46] Shannon P, Markiel A, Ozier O, Baliga NS, Wang JT (2003). Cytoscape: a software environment for integrated models of biomolecular interaction networks.. Genome Res.

