# Supplementary figures and images for: Using Regulatory and Epistatic Networks to Extend the Findings of a Genome Scan: Identifying the Gene Drivers of Pigmentation in Merino Sheep
Source: PLoS One. 2011 Jun 20;6(6):e21158. doi: 10.1371/journal.pone.0021158 (PMC3119053; doi:10.1371/journal.pone.0021158)

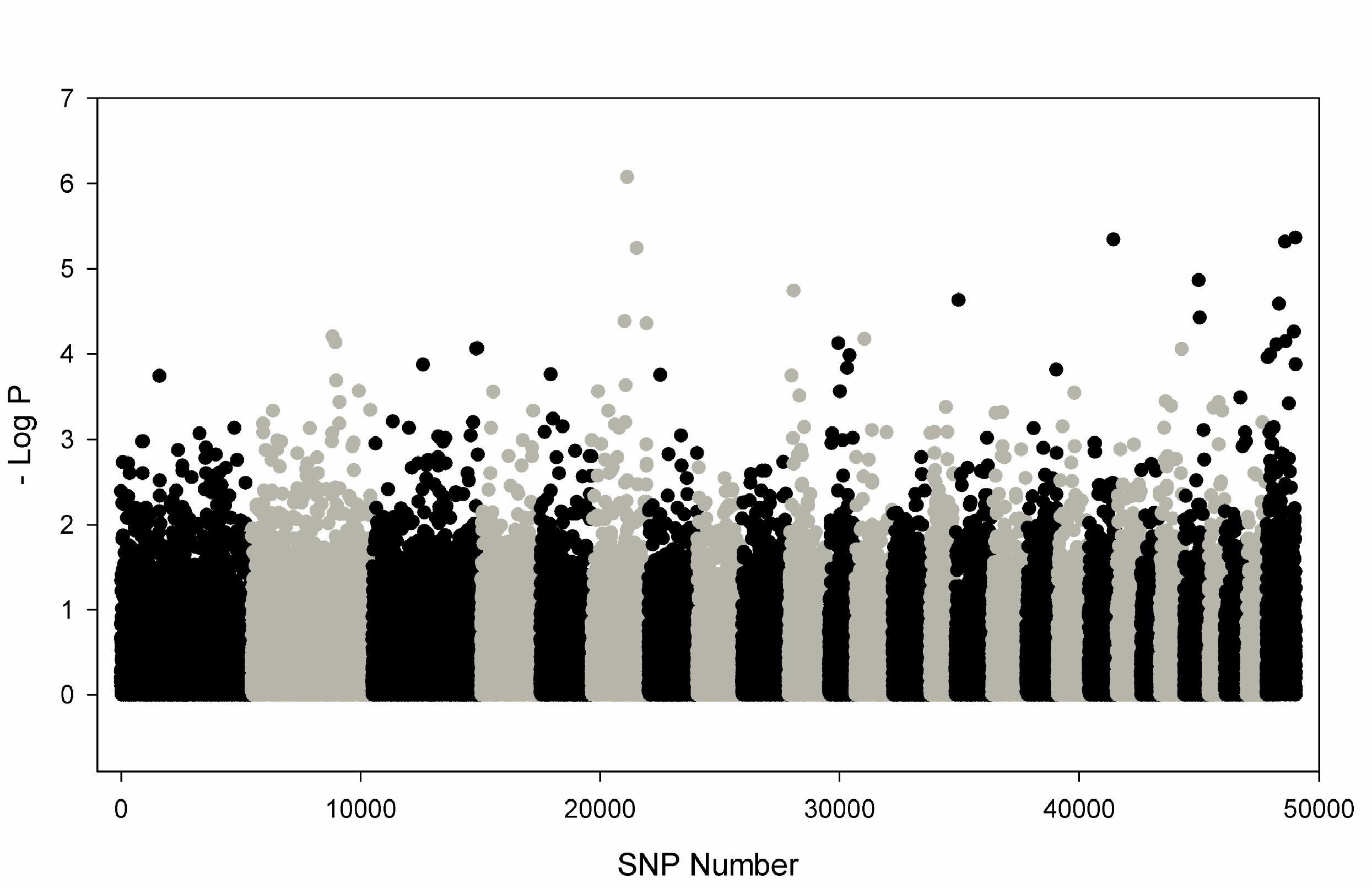

Supplement: Figure S1 — Genome wide association results for piebald. The strength of association expressed as negative log P values (Y axis) are shown for 49034 SNP (X axis) arranged in genomic order from chromosome 1 (far left) to the X chromosome (far right). (TIF) [file pone.0021158.s001.tif]

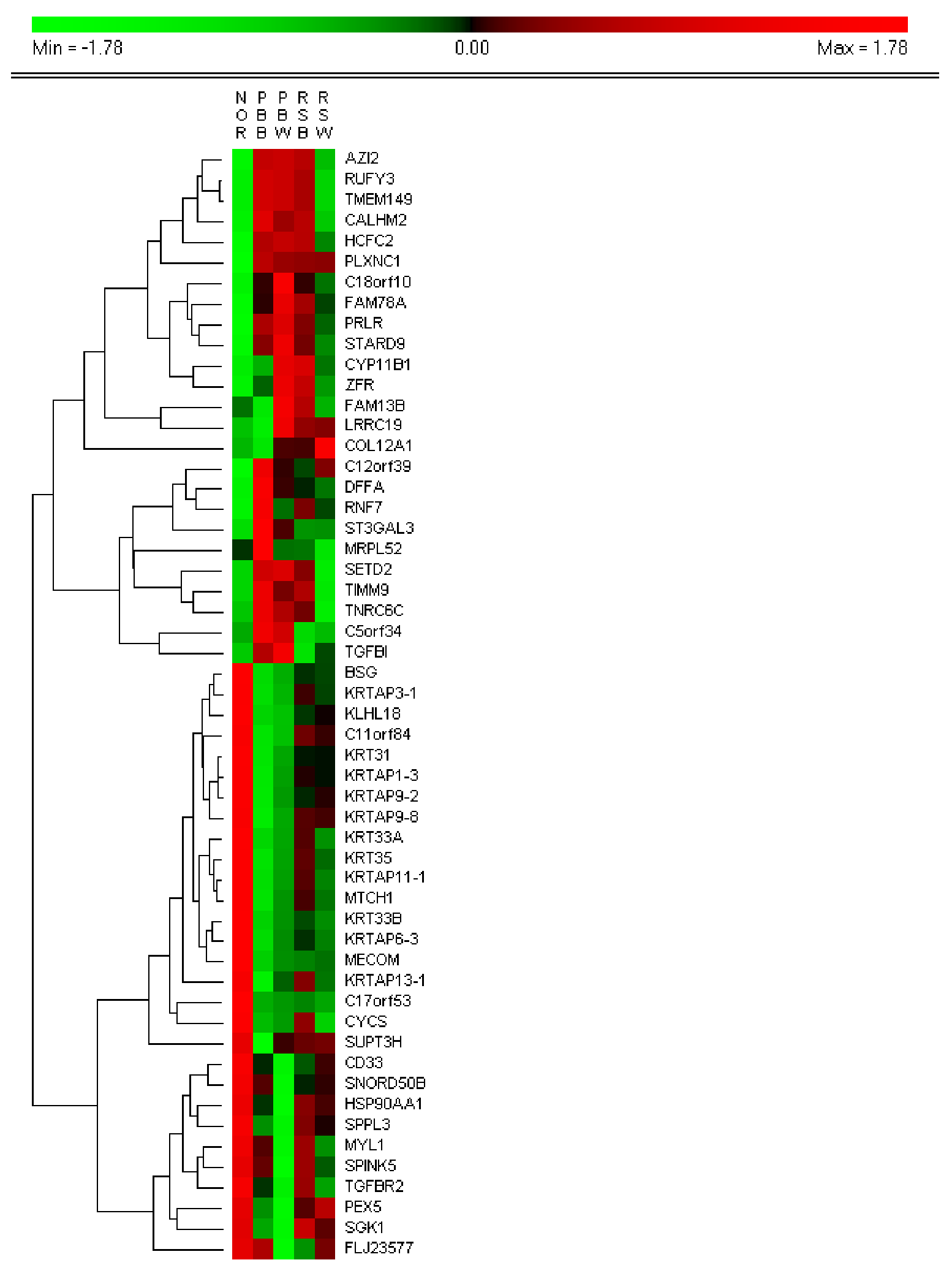

Supplement: Figure S2 — Co-regulation of 54 genes associated with pigmentation. Gene expression was examined using five tissue types as follows: white skin tissue from a non-pigmented animal (NOR); black skin tissue from a piebald animal (PBB); white skin tissue from a piebald animal (PBW); black skin tissue from a self color black animal (RSB) and white skin tissue from a self color black animal (RSW). The normalised mean expression (NME) within each tissue type is represented using color ranging from green (down regulation) through to red (up regulation). The 54 genes displayed were differentially expressed in at least 4 of the 7 tissue type contrasts examined (Figure 1 describes the 7 contrasts). Hierarchical clustering was performed to identify genes which are expressed in a coordinated way across tissue types, which revealed a set of 11 keratin genes strongly down regulated in piebald tissue. (TIF) [file pone.0021158.s002.tif]

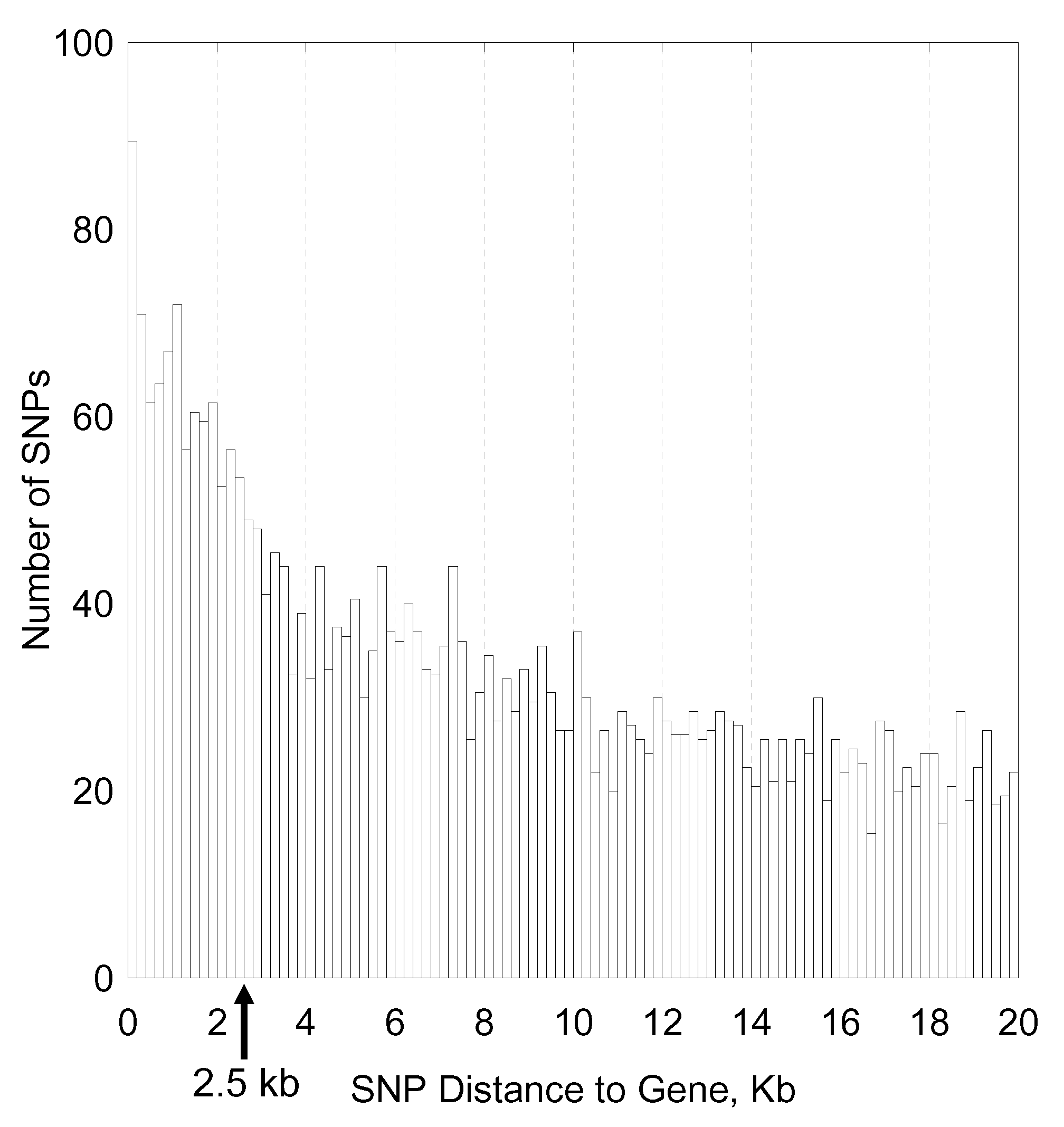

Supplement: Figure S3 — Physical proximity of SNP on the ovine SNP50 BeadChip to genes annotated in Ovine Genome Assembly v1.0 (https://www.biolives.csiro.au/cgi-bin/gbrowse/oar1.0/). A total of 47,275 SNP with known base pair location were examined. Of these, 15,624 SNP (or 33%) were intragenic while 24,921 (53%) were located greater than 20 Kb from the nearest gene. The distribution of the remaining 6,730 (14%) of SNP is shown where the SNP to gene distance was binned in increments of 0.2 Kb from 1 bp to 20 Kb. Importantly, the analysis reported 17,223 SNP were located with 2.5 Kb of the nearest gene which was our empirical threshold for defining SNP as potentially be cis-acting. (TIF) [file pone.0021158.s003.tif]

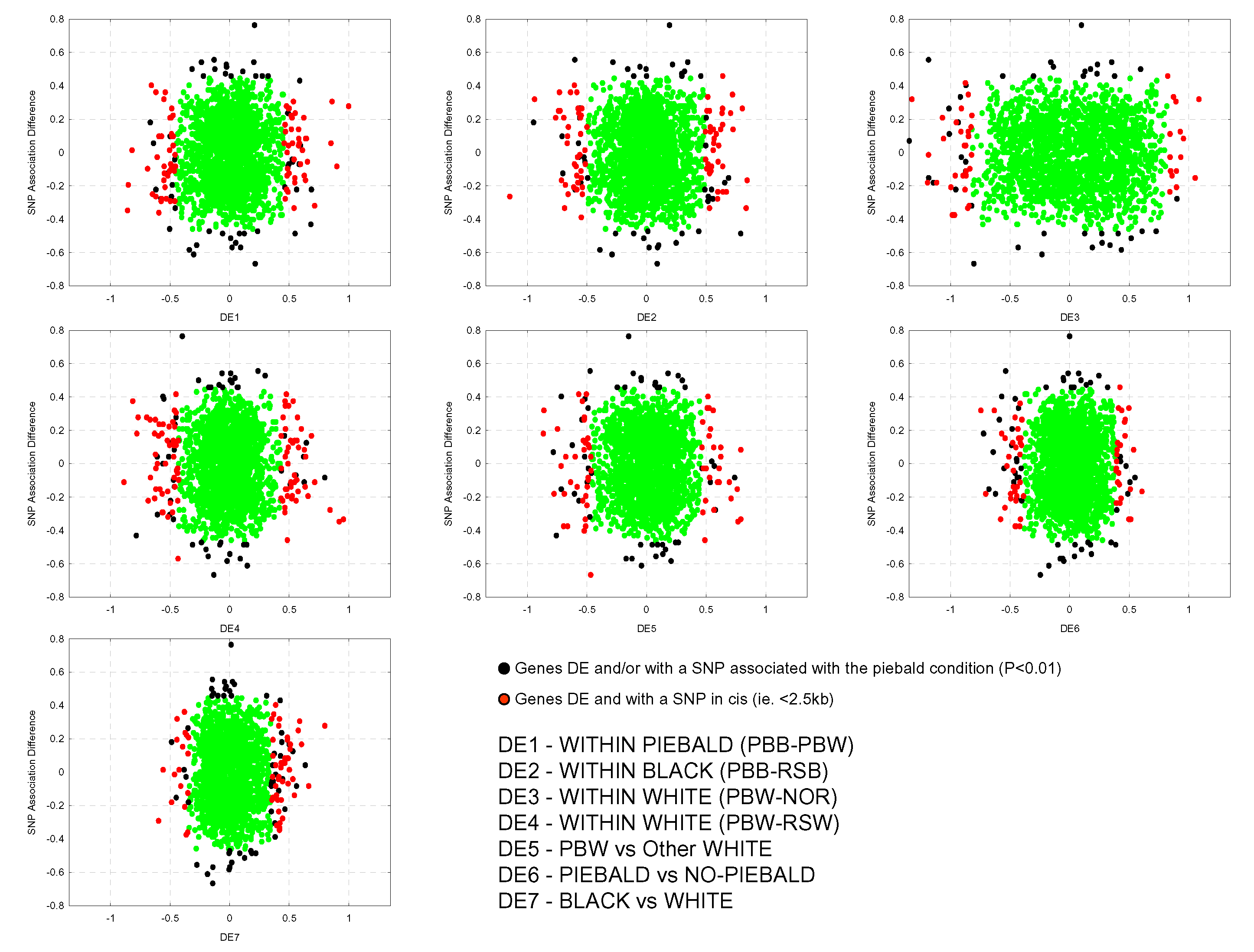

Supplement: Figure S4 — Plot of allele frequency difference versus gene expression for a set of 1,935 genes. For each of seven gene expression contrasts (termed DE1–DE7; refer to the materials and methods), the position of each symbol plots both the SNP allele frequency difference between piebald and non-piebald animals (Y axis) and differential gene expression (X axis). Red symbols represent genes which were both differentially expressed (piebald versus normal) and have a SNP within 2.5 Kb. Black symbols represent genes which either (i) displayed differential expression (p-value<0.05) or (ii) are located within 1 Mb of an associated SNP (p-value<0.01). The remaining green symbols represent genes which were neither differentially expressed nor located near associated SNP. (TIF) [file pone.0021158.s004.tif]

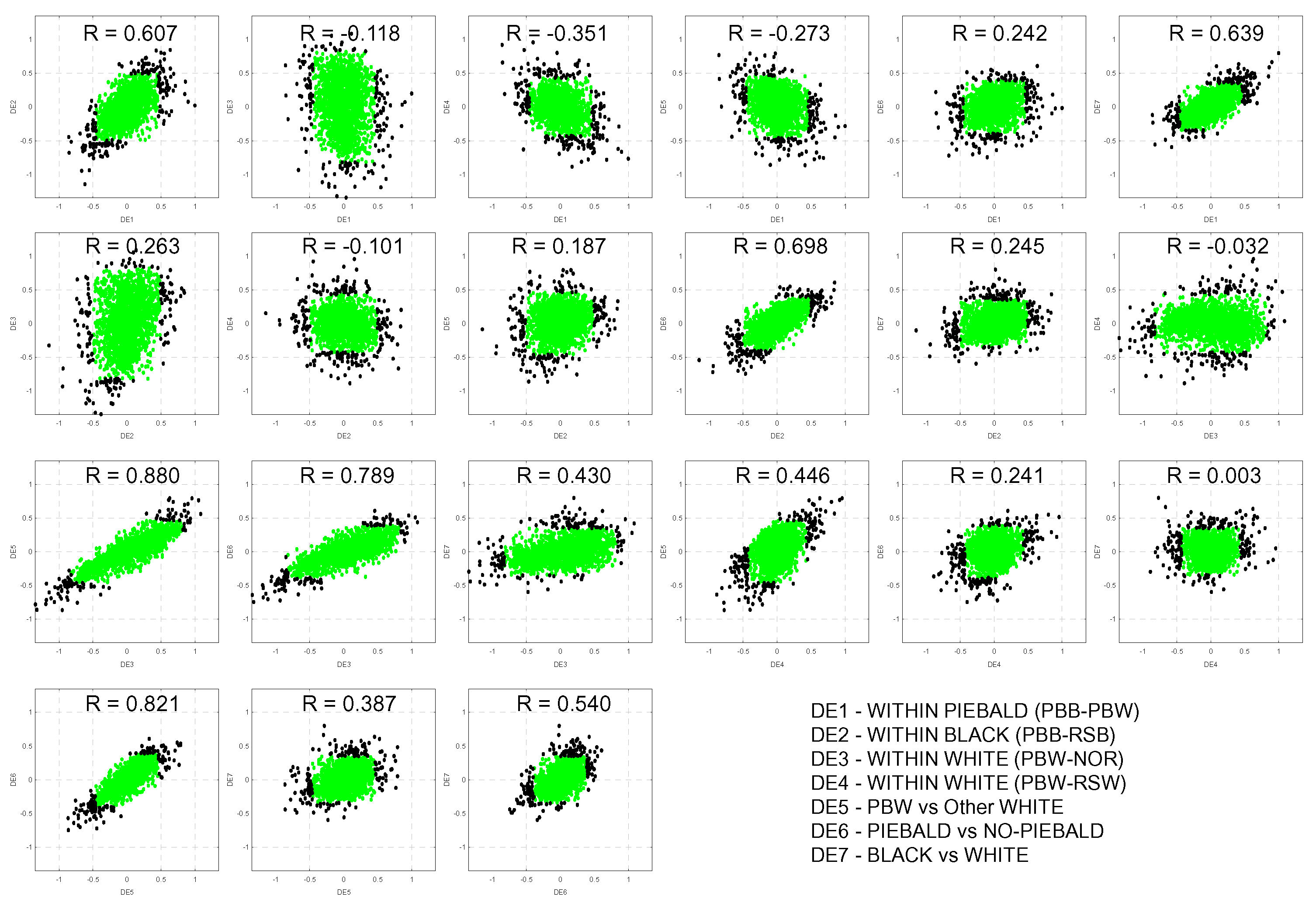

Supplement: Figure S5 — Correlation of differential expression for 1,935 genes. Differential gene expression observed within each of seven contrasts (termed DE1–DE7) were correlated in 21 pair-wise comparisons. The highest correlation (R = 0.88) was observed between DE3 and DE5, both of which examined the difference between piebald and non-piebald tissues. The only difference between the two contrasts being inclusion of an additional tissue type (RSW) in DE5. Similarly, the second and third highest correlations (DE5 and DE6 R = 0.82; DE3 and DE6 R = 0.79) was also found between contrasts constructed between piebald and non-piebald tissue types. Three contrasts together (DE3, DE5 and DE6) were used to assign genes as either over-expressed (red), under-expressed (green) or having unchanged expression (orange) in the gene networks relating to piebald (Figure 3). (TIF) [file pone.0021158.s005.tif]

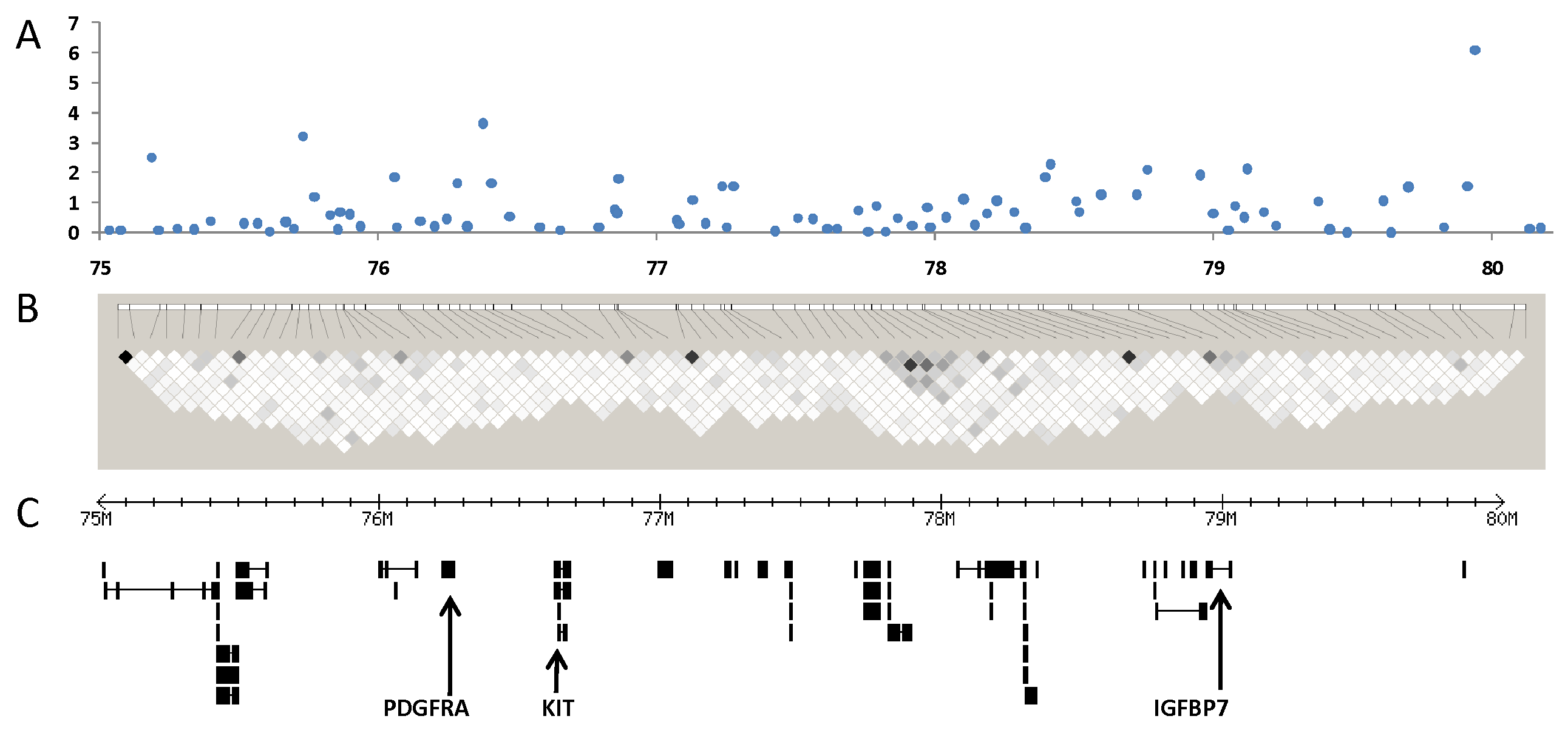

Supplement: Figure S6 — SNP association results for OAR6. (A) The strength of association between each SNP and piebald is given as negative log P values (X axis) across a 5 Mb region of sheep chromosome (OAR) 6 (X axis). This shows the position of the highest ranked SNP (s49104), genome wide, at Mb position 79.9. (B) Pair-wise linkage disequilibrium between SNP, measured as r 2, was calculated using all 96 animals (24 cases and 72 controls) and plotted as a heatmap in Haploview. This shows LD extends for only short distances across the region. The relative location of each annotated gene within the region is shown in (C). (TIF) [file pone.0021158.s006.tif]
