# Supplementary material for: Using Regulatory and Epistatic Networks to Extend the Findings of a Genome Scan: Identifying the Gene Drivers of Pigmentation in Merino Sheep
Source: PLoS One. 2011 Jun 20;6(6):e21158. doi: 10.1371/journal.pone.0021158 (PMC3119053; doi:10.1371/journal.pone.0021158)
Supplement: Table S1 — Piebald associated genes. All of the genes listed displayed either DE in one or more contrasts and a cis-SNP or the absence of DE but an associated SNP within the chromosomal region (<1 Mb). (DOC) [file pone.0021158.s007.doc]

Table S1

|  |  |  |  |  |  |  |  |  |  |  |  |
| --- | --- | --- | --- | --- | --- | --- | --- | --- | --- | --- | --- |
| **Gene_ID** | **Mean Expression for each variety** | | | | | **DE** | **SNP** | **Distance to** | **Genotype** | **Frequency in** | **Frequency in** |
|  | **NOR** | **PBB** | **PBW** | **RSB** | **RSW** |  |  | **gene (Kb)** | **difference** | **Piebald** | **Non-Piebald** |
| ABCA4 | 3.96 | 2.77 | 3.40 | 4.02 | 4.66 | 0001000 | s53104 | 26.3 | -0.319 | 0.60 | 0.76 |
| ACIN1 | 5.77 | 7.24 | 6.59 | 6.60 | 6.65 | 1100000 | OAR7_22306460 | 0.0 | 0.139 | 0.56 | 0.49 |
| ACOX1 | 7.74 | 6.68 | 6.20 | 7.17 | 7.05 | 0000100 | OAR11_58566699 | 0.0 | -0.403 | 0.35 | 0.56 |
| ACOX2 | 2.72 | 3.78 | 4.76 | 3.51 | 3.56 | 0001000 | OAR19_45112987 | 0.0 | -0.167 | 0.48 | 0.56 |
| ADAMTS2 | 4.14 | 5.28 | 4.84 | 4.71 | 5.93 | 1001000 | s19957 | 0.0 | 0.306 | 0.71 | 0.56 |
| ADK | 10.62 | 9.01 | 9.05 | 9.73 | 9.67 | 0010110 | OAR25_31766050 | 0.0 | -0.375 | 0.46 | 0.65 |
| AFF1 | 7.10 | 6.63 | 6.97 | 7.91 | 7.30 | 0100000 | s23686 | 0.0 | -0.222 | 0.42 | 0.53 |
| AGPAT4 | 5.46 | 5.59 | 6.65 | 4.42 | 6.70 | 0100001 | s67174 | 0.0 | 0.236 | 0.71 | 0.59 |
| AGTPBP1 | 5.93 | 8.09 | 7.49 | 7.25 | 8.55 | 1100000 | OAR2_35234270 | 0.0 | -0.292 | 0.42 | 0.56 |
| AKAP6 | 10.39 | 9.23 | 9.56 | 9.55 | 10.05 | 0000001 | OAR18_44540786 | 0.0 | 0.222 | 0.56 | 0.45 |
| ALPK1 | 8.81 | 5.84 | 7.86 | 8.97 | 8.46 | 1100010 | OAR6_15819645 | 3.2 | 0.319 | 0.77 | 0.61 |
| ANK3 | 12.86 | 9.93 | 11.05 | 11.76 | 10.76 | 0110010 | OAR25_16148956 | 0.0 | -0.167 | 0.44 | 0.52 |
| ANKRD10 | 3.25 | 4.31 | 3.93 | 3.96 | 3.58 | 0000001 | s35682 | 0.0 | -0.042 | 0.71 | 0.73 |
| ANKRD13C | 2.88 | 3.97 | 4.53 | 3.86 | 4.24 | 0100000 | OAR1_47449016 | 0.2 | -0.083 | 0.42 | 0.46 |
| ANKRD28 | 7.46 | 7.23 | 5.98 | 6.45 | 7.35 | 1000100 | OAR1_293382896 | 0.0 | 0.139 | 0.73 | 0.66 |
| ANTXR2 | 6.47 | 8.68 | 8.42 | 7.33 | 8.49 | 1100010 | OAR6_103247069 | 0.0 | -0.194 | 0.58 | 0.68 |
| AP2A2 | 7.39 | 7.02 | 8.16 | 7.68 | 7.30 | 0001000 | s58433 | 0.0 | 0.000 | 0.56 | 0.56 |
| API5 | 4.35 | 5.24 | 6.23 | 5.86 | 6.42 | 0000000 | s71472 | 0.0 | 0.472 | 0.88 | 0.64 |
| ARHGEF12 | 4.28 | 2.78 | 5.12 | 4.90 | 4.07 | 1100000 | OAR15_32026400 | 0.0 | -0.250 | 0.54 | 0.67 |
| ARMC8 | 8.69 | 7.02 | 7.91 | 8.59 | 8.65 | 0000010 | OAR1_268926215 | 0.0 | -0.194 | 0.79 | 0.89 |
| ARSK | 5.77 | 7.87 | 7.79 | 6.81 | 7.69 | 0100010 | OAR5_100495456 | 0.0 | 0.250 | 0.79 | 0.67 |
| ASPH | 9.51 | 7.44 | 9.02 | 9.44 | 9.05 | 1100010 | s71803 | 0.0 | -0.167 | 0.58 | 0.67 |
| ASPSCR1 | 11.18 | 9.41 | 9.64 | 10.34 | 10.32 | 0010000 | s15499 | 0.0 | 0.097 | 0.85 | 0.81 |
| ATP1A1 | 7.30 | 7.51 | 7.84 | 6.90 | 7.13 | 0100000 | s10849 | 106.4 | -0.278 | 0.46 | 0.60 |
| ATP6V0A4 | 6.49 | 7.83 | 8.27 | 8.26 | 9.17 | 0000000 | s57909 | 0.7 | 0.083 | 0.58 | 0.54 |
| ATP8B1 | 2.91 | 2.88 | 5.45 | 4.66 | 4.66 | 1100000 | OAR23_60895236 | 26.8 | 0.208 | 0.60 | 0.50 |
| ATRN | 6.15 | 8.43 | 8.42 | 7.59 | 8.99 | 0010010 | OAR13_55578882 | 0.0 | 0.458 | 0.67 | 0.44 |
| ATRX | 6.77 | 8.62 | 8.37 | 7.67 | 8.74 | 0100010 | OARX_5584397 | 0.0 | 0.014 | 0.90 | 0.89 |
| ATXN7 | 7.84 | 7.09 | 7.85 | 7.86 | 8.36 | 0001000 | s57682 | 0.1 | -0.292 | 0.58 | 0.73 |
| AZI2 | 3.84 | 6.01 | 6.31 | 6.13 | 4.73 | 0011111 | s53660 | 0.0 | 0.000 | 0.85 | 0.85 |
| BATF3 | 2.58 | 4.75 | 4.73 | 4.78 | 5.02 | 0000100 | OAR12_75778746 | 0.6 | 0.097 | 0.75 | 0.70 |
| BIRC6 | 4.98 | 4.12 | 5.90 | 6.26 | 5.81 | 0100000 | s29979 | 12.4 | 0.222 | 0.90 | 0.78 |
| BMF | 10.43 | 9.38 | 9.54 | 10.05 | 10.15 | 0010100 | OAR7_36956463 | 0.0 | -0.083 | 0.65 | 0.69 |
| BSND | 8.09 | 6.94 | 8.55 | 6.82 | 8.12 | 0000001 | s43731 | 0.0 | -0.014 | 0.83 | 0.84 |
| BTC | 2.57 | 2.78 | 5.09 | 3.64 | 2.84 | 1001100 | OAR6_97962553 | 0.0 | 0.222 | 0.79 | 0.68 |
| BTN3A2 | 2.66 | 4.07 | 2.99 | 3.42 | 3.03 | 1000001 | s13172 | 0.0 | 0.014 | 0.56 | 0.56 |
| C1GALT1C1 | 5.87 | 6.07 | 7.29 | 7.13 | 7.01 | 0000000 | OARX_126032756 | 112.6 | -0.583 | 0.63 | 0.92 |
| C1QTNF2 | 7.23 | 6.81 | 8.66 | 8.40 | 8.67 | 1100000 | OAR5_76042662 | 0.0 | 0.264 | 0.71 | 0.58 |
| CABP2 | 3.25 | 3.75 | 4.84 | 3.83 | 4.06 | 1000000 | s00044 | 0.0 | -0.292 | 0.71 | 0.85 |
| CAMK2A | 5.29 | 6.89 | 6.73 | 6.40 | 7.83 | 0001000 | s29856 | 0.0 | -0.028 | 0.69 | 0.70 |
| CAPN2 | 2.62 | 3.39 | 3.19 | 4.14 | 4.03 | 0001000 | s35352 | 0.0 | -0.056 | 0.75 | 0.78 |
| CAV1 | 5.23 | 3.98 | 6.07 | 5.00 | 5.38 | 1000000 | OAR4_54885111 | 0.0 | -0.111 | 0.81 | 0.87 |
| CBARA1 | 4.11 | 4.81 | 5.96 | 5.09 | 5.02 | 1001100 | OAR25_29718439 | 0.0 | 0.042 | 0.52 | 0.50 |
| CCDC109B | 5.78 | 7.36 | 7.83 | 5.72 | 8.17 | 0100010 | OAR6_18823493 | 0.0 | -0.056 | 0.88 | 0.90 |
| CCDC88A | 6.07 | 7.63 | 8.04 | 6.99 | 8.88 | 0010000 | OAR3_72422787 | 0.0 | 0.153 | 0.67 | 0.59 |
| CCNT2 | 4.05 | 5.35 | 5.60 | 6.01 | 6.72 | 0000001 | OAR2_185259305 | 0.0 | 0.194 | 0.85 | 0.76 |
| CD34 | 7.25 | 5.89 | 7.65 | 7.56 | 7.52 | 1000000 | OAR12_81101401 | 0.0 | 0.056 | 0.56 | 0.53 |
| CD9 | 9.63 | 9.32 | 9.01 | 8.33 | 9.58 | 0000000 | s65742 | 16.6 | -0.569 | 0.29 | 0.58 |
| CDKL2 | 3.23 | 4.51 | 3.90 | 4.65 | 3.29 | 0000001 | OAR6_98813599 | 0.0 | 0.250 | 0.83 | 0.71 |
| CDYL | 2.49 | 3.11 | 4.45 | 4.65 | 3.94 | 1100100 | OAR20_52759557 | 0.7 | 0.403 | 0.79 | 0.59 |
| CHD7 | 8.51 | 7.16 | 7.00 | 7.65 | 8.03 | 0001100 | s12020 | 0.0 | -0.333 | 0.58 | 0.75 |
| CLASP2 | 6.27 | 6.47 | 7.48 | 6.57 | 8.17 | 0001000 | OAR19_8093302 | 0.0 | -0.167 | 0.58 | 0.67 |
| COL12A1 | 2.08 | 2.09 | 3.54 | 3.50 | 4.62 | 1101001 | s29054 | 0.0 | 0.111 | 0.81 | 0.76 |
| COL5A3 | 6.58 | 3.56 | 5.46 | 5.15 | 5.71 | 1000001 | s26328 | 16.3 | -0.292 | 0.42 | 0.56 |
| CORO1A | 5.83 | 6.78 | 7.93 | 6.49 | 6.70 | 0001100 | OAR24_28699670 | 1.0 | 0.208 | 1.00 | 0.90 |
| CORO1C | 3.97 | 4.34 | 4.04 | 4.67 | 5.87 | 0001100 | s51903 | 0.0 | 0.375 | 0.63 | 0.44 |
| CPD | 7.99 | 8.83 | 8.60 | 7.67 | 8.74 | 0100000 | s42127 | 0.0 | -0.069 | 0.65 | 0.68 |
| CPEB2 | 8.73 | 8.82 | 8.75 | 8.18 | 9.08 | 0000000 | OAR6_124676719 | 0.0 | 0.486 | 0.73 | 0.49 |
| CREB5 | 7.79 | 6.44 | 6.46 | 6.75 | 7.92 | 0000110 | OAR4_71639773 | 0.0 | -0.333 | 0.42 | 0.58 |
| CSRP2 | 8.10 | 6.79 | 6.01 | 7.45 | 7.75 | 0010110 | OAR3_120050967_X | 0.0 | -0.347 | 0.48 | 0.65 |
| CTDSPL | 4.19 | 6.54 | 5.93 | 5.48 | 5.78 | 1000001 | OAR19_11623353 | 0.0 | 0.278 | 0.67 | 0.53 |
| CTSK | 10.27 | 9.53 | 9.96 | 9.03 | 10.13 | 0000001 | OAR1_106946819 | 0.0 | 0.014 | 0.69 | 0.68 |
| CUTC | 3.37 | 5.07 | 4.03 | 4.69 | 4.26 | 1000001 | OAR22_23763191 | 0.0 | 0.083 | 0.81 | 0.77 |
| CYCS | 13.65 | 10.38 | 11.43 | 12.24 | 11.36 | 0110110 | OAR2_236658998 | 7.6 | -0.361 | 0.40 | 0.58 |
| DAAM1 | 7.61 | 8.20 | 8.63 | 7.38 | 8.72 | 0000000 | OAR7_75022469 | 0.0 | 0.472 | 0.88 | 0.64 |
| DCBLD2 | 5.07 | 6.81 | 7.55 | 6.24 | 7.83 | 0001000 | OAR1_174387395 | 10.6 | -0.125 | 0.69 | 0.75 |
| DCLK1 | 8.80 | 7.64 | 7.95 | 8.85 | 8.06 | 0100010 | s16219 | 0.0 | 0.264 | 0.85 | 0.72 |
| DCTN5 | 12.21 | 10.66 | 10.56 | 11.47 | 11.51 | 0010110 | s25750 | 0.0 | 0.125 | 0.67 | 0.60 |
| DCUN1D1 | 7.65 | 7.44 | 8.60 | 8.31 | 8.54 | 1000000 | OAR1_218477867 | 0.0 | 0.097 | 0.58 | 0.53 |
| DDR2 | 12.13 | 10.64 | 10.93 | 11.78 | 11.85 | 0000110 | OAR1_121390041 | 0.0 | -0.194 | 0.52 | 0.62 |
| DDX39 | 2.02 | 0.72 | 1.82 | 2.08 | 1.52 | 0100000 | s45523 | 0.0 | -0.014 | 0.58 | 0.59 |
| DDX47 | 5.44 | 7.18 | 7.22 | 6.47 | 7.86 | 1000000 | OAR3_217297404 | 0.0 | 0.125 | 0.73 | 0.67 |
| DEF6 | 10.65 | 9.34 | 9.25 | 9.89 | 9.96 | 0010100 | OAR20_9513381 | 0.0 | -0.097 | 0.58 | 0.63 |
| DENND2D | 4.71 | 4.80 | 6.43 | 6.01 | 5.27 | 1001100 | s66406 | 0.0 | 0.097 | 0.67 | 0.62 |
| DGUOK | 5.43 | 5.90 | 6.34 | 6.50 | 5.88 | 0000100 | s68643 | 0.0 | -0.028 | 0.85 | 0.87 |
| DMD | 5.11 | 6.47 | 7.47 | 6.14 | 8.10 | 0001000 | s13796 | 0.0 | -0.250 | 0.58 | 0.71 |
| DOCK5 | 4.42 | 6.23 | 5.94 | 6.64 | 6.57 | 0000001 | OAR2_41879685 | 0.0 | 0.167 | 0.79 | 0.71 |
| DOCK7 | 2.47 | 4.18 | 4.36 | 4.67 | 4.18 | 0000001 | OAR1_38581202 | 0.0 | -0.153 | 0.67 | 0.74 |
| DSPP | 8.86 | 8.23 | 8.12 | 8.72 | 9.24 | 1100010 | s18093 | 0.0 | 0.056 | 0.85 | 0.83 |
| DYNLRB1 | 9.17 | 8.02 | 7.82 | 8.67 | 8.84 | 0010110 | OAR13_68535533 | 0.0 | 0.125 | 0.58 | 0.52 |
| EFNA5 | 8.44 | 9.15 | 8.91 | 8.59 | 9.15 | 0000000 | s72651 | 0.0 | -0.417 | 0.58 | 0.79 |
| EHD3 | 11.63 | 10.32 | 10.93 | 11.24 | 11.28 | 0000100 | s48847 | 0.0 | 0.042 | 0.69 | 0.67 |
| EIF3B | 3.84 | 3.89 | 4.19 | 3.56 | 2.74 | 0001001 | OAR24_44025537 | 2.4 | 0.167 | 0.94 | 0.85 |
| EIF3CL | 7.63 | 8.65 | 8.77 | 8.29 | 9.14 | 0000000 | s68253 | 0.0 | -0.542 | 0.31 | 0.58 |
| EIF5B | 9.88 | 9.17 | 9.68 | 9.02 | 9.62 | 0010110 | OAR3_108365529 | 0.0 | -0.139 | 0.56 | 0.63 |
| ELP4 | 5.88 | 5.78 | 4.98 | 5.94 | 5.44 | 1000000 | OAR15_65969620 | 0.0 | -0.222 | 0.42 | 0.53 |
| EPB41L4B | 8.14 | 7.87 | 8.16 | 8.33 | 7.97 | 1001000 | OAR2_13411521 | 0.0 | -0.139 | 0.56 | 0.63 |
| EXOC5 | 7.24 | 5.85 | 7.28 | 7.83 | 6.66 | 0001000 | OAR7_73093180 | 0.0 | -0.194 | 0.46 | 0.56 |
| EXOSC9 | 6.11 | 8.28 | 7.74 | 7.75 | 8.33 | 1000001 | OAR6_5533333 | 0.0 | -0.014 | 0.63 | 0.63 |
| FABP9 | 10.49 | 9.76 | 9.98 | 9.74 | 10.11 | 0000000 | OAR9_60512150 | 0.0 | 0.458 | 0.73 | 0.50 |
| FAT2 | 3.45 | 4.93 | 5.39 | 4.38 | 5.39 | 0000100 | s48255 | 0.0 | -0.083 | 0.63 | 0.67 |
| FBN1 | 9.69 | 8.60 | 8.44 | 8.76 | 7.79 | 0001000 | OAR7_64793910 | 12.3 | 0.278 | 0.79 | 0.65 |
| FBXL20 | 5.51 | 5.92 | 6.28 | 6.37 | 6.86 | 1000000 | OAR11_41816861 | 0.0 | 0.014 | 0.88 | 0.87 |
| FCHSD2 | 5.98 | 7.52 | 8.27 | 7.62 | 8.29 | 0010100 | OAR15_55438774 | 2.5 | 0.333 | 0.63 | 0.46 |
| FERMT1 | 5.89 | 6.36 | 6.68 | 6.27 | 5.55 | 0001000 | OAR13_51167861 | 309.7 | 0.347 | 0.79 | 0.62 |
| FGFR1OP | 7.87 | 6.73 | 7.28 | 6.75 | 7.09 | 1000000 | OAR8_95443730 | 0.0 | 0.167 | 0.90 | 0.81 |
| FHL1 | 7.86 | 8.69 | 8.65 | 8.54 | 8.99 | 0100010 | OARX_115665260 | 0.0 | -0.125 | 0.85 | 0.92 |
| FKBP3 | 4.86 | 7.87 | 8.41 | 6.11 | 8.02 | 0100010 | OAR18_57583120 | 0.5 | 0.167 | 0.88 | 0.79 |
| FLT1 | 8.02 | 7.42 | 8.14 | 8.88 | 8.51 | 0100000 | OAR10_32276024 | 3.3 | -0.153 | 0.52 | 0.60 |
| FRMPD3 | 1.52 | 4.00 | 2.57 | 3.61 | 2.94 | 1000001 | s69242 | 3.7 | 0.306 | 0.65 | 0.49 |
| FRY | 6.78 | 8.50 | 8.19 | 7.97 | 8.81 | 0000000 | OAR10_29223007 | 0.0 | 0.528 | 0.69 | 0.42 |
| FUBP3 | 5.42 | 6.28 | 5.46 | 5.00 | 6.24 | 1100000 | s12025 | 0.0 | -0.014 | 0.69 | 0.69 |
| GAB1 | 5.21 | 7.49 | 7.48 | 7.05 | 8.02 | 0010000 | OAR17_15846928 | 0.0 | -0.139 | 0.83 | 0.90 |
| GABPB1 | 10.01 | 7.93 | 8.73 | 9.27 | 9.52 | 0000110 | OAR1_107239370 | 0.0 | -0.042 | 0.79 | 0.81 |
| GABRB1 | 9.71 | 8.26 | 8.02 | 9.26 | 9.30 | 0010110 | OAR6_71981745 | 47.7 | -0.375 | 0.40 | 0.58 |
| GATAD2A | 5.28 | 6.18 | 5.77 | 6.35 | 7.25 | 0001000 | s22781 | 0.0 | -0.181 | 0.60 | 0.69 |
| GDPD5 | 5.20 | 5.87 | 6.36 | 6.10 | 7.83 | 0001000 | s31847 | 0.0 | 0.181 | 0.85 | 0.76 |
| GLI3 | 5.85 | 7.48 | 7.58 | 6.81 | 8.39 | 0001000 | OAR4_83976143 | 0.0 | -0.236 | 0.67 | 0.78 |
| GNE | 3.61 | 2.92 | 3.80 | 3.88 | 2.74 | 0101000 | OAR2_55617004 | 0.0 | -0.069 | 0.81 | 0.85 |
| GPATCH1 | 6.39 | 5.64 | 7.35 | 7.33 | 8.00 | 0100000 | s40481 | 0.0 | 0.056 | 0.98 | 0.95 |
| GPBP1 | 7.03 | 7.77 | 8.19 | 7.25 | 8.16 | 0100000 | OAR16_24177015 | 0.0 | -0.069 | 0.83 | 0.87 |
| GSDMC | 3.09 | 3.82 | 5.26 | 5.49 | 4.90 | 0100000 | s34860 | 2.2 | -0.278 | 0.42 | 0.56 |
| GSR | 4.41 | 4.87 | 5.27 | 4.46 | 4.74 | 0100010 | OAR26_29597675 | 0.0 | -0.236 | 0.58 | 0.70 |
| HAPLN1 | 5.02 | 5.09 | 6.57 | 6.13 | 6.56 | 1100000 | OAR5_88862060 | 0.0 | 0.069 | 0.77 | 0.74 |
| HBEGF | 5.90 | 6.30 | 5.30 | 6.49 | 5.17 | 1000001 | OAR5_53183086 | 0.0 | 0.208 | 0.92 | 0.81 |
| HCFC2 | 5.72 | 7.38 | 7.96 | 7.42 | 7.19 | 0010111 | OAR3_185518285 | 0.0 | -0.028 | 0.90 | 0.91 |
| HIPK1 | 10.05 | 8.83 | 8.99 | 9.42 | 9.44 | 0010010 | OAR1_96072427 | 0.0 | -0.111 | 0.46 | 0.51 |
| HISPPD1 | 3.57 | 3.80 | 3.20 | 4.35 | 4.66 | 0001000 | OAR5_108231774 | 0.0 | -0.028 | 0.50 | 0.51 |
| HM13 | 5.79 | 7.62 | 7.10 | 6.33 | 6.98 | 0000010 | s70152 | 0.0 | 0.056 | 0.94 | 0.91 |
| HMGCR | 6.67 | 5.97 | 5.20 | 5.65 | 5.52 | 0000100 | s63040 | 0.2 | -0.250 | 0.50 | 0.63 |
| HSPA5 | 5.52 | 6.19 | 6.67 | 7.01 | 7.89 | 0001000 | OAR3_11261007 | 0.7 | -0.097 | 0.52 | 0.57 |
| IGFBP7 | 11.37 | 10.35 | 9.70 | 9.87 | 10.70 | 0000100 | s49104 | 915.2 | -0.667 | 0.54 | 0.88 |
| IL1R2 | 8.21 | 6.33 | 7.91 | 7.03 | 6.19 | 0001000 | s49870 | 3.6 | 0.139 | 0.50 | 0.43 |
| INPP4B | 1.43 | 4.46 | 2.83 | 4.61 | 3.97 | 1001001 | OAR17_16676148 | 185.3 | 0.278 | 0.71 | 0.57 |
| KANK1 | 8.80 | 7.46 | 8.63 | 8.77 | 9.29 | 0100000 | OAR2_73211287 | 0.0 | -0.250 | 0.60 | 0.73 |
| KCNMB1 | 4.92 | 5.35 | 7.66 | 5.98 | 6.31 | 1001000 | s11386 | 0.0 | -0.097 | 0.81 | 0.86 |
| KIF23 | 5.51 | 6.27 | 5.60 | 5.94 | 5.36 | 0000001 | s22365 | 12.2 | -0.333 | 0.50 | 0.67 |
| KLF4 | 10.94 | 9.90 | 9.63 | 9.91 | 9.92 | 0010000 | OAR2_14584156 | 491.6 | 0.264 | 0.96 | 0.83 |
| KLHL18 | 11.45 | 8.83 | 9.16 | 9.94 | 10.14 | 0010111 | OAR19_55177848 | 0.0 | 0.319 | 0.73 | 0.57 |
| KLHL21 | 10.50 | 9.89 | 10.01 | 10.01 | 10.45 | 0000001 | OAR12_49815403 | 0.0 | -0.278 | 0.58 | 0.72 |
| KLK10 | 12.38 | 11.19 | 11.50 | 12.11 | 12.22 | 0000010 | s26891 | 0.0 | 0.056 | 0.50 | 0.47 |
| KRT31 | 14.06 | 11.25 | 12.11 | 12.77 | 12.57 | 0110111 | OAR11_43737226 | 0.0 | 0.069 | 0.71 | 0.67 |
| KRT85 | 10.17 | 8.75 | 8.82 | 9.43 | 9.54 | 0010000 | s25197 | 0.0 | -0.014 | 0.73 | 0.74 |
| KRTCAP2 | 6.80 | 8.21 | 8.50 | 8.04 | 8.66 | 0010000 | s24278 | 0.0 | 0.125 | 0.79 | 0.73 |
| LARP1 | 7.38 | 7.14 | 8.10 | 7.99 | 7.13 | 0001000 | s01547 | 0.0 | -0.111 | 0.50 | 0.56 |
| LARP7 | 6.29 | 7.99 | 7.71 | 6.51 | 8.76 | 1100010 | s14758 | 64.3 | -0.431 | 0.46 | 0.67 |
| LIN28B | 4.87 | 5.45 | 6.52 | 6.54 | 5.72 | 0001000 | s20065 | 0.0 | -0.153 | 0.83 | 0.91 |
| LMBRD1 | 6.96 | 8.87 | 9.03 | 8.87 | 9.43 | 0000001 | s69406 | 15.5 | -0.306 | 0.52 | 0.67 |
| LOC528939 | 4.86 | 4.55 | 4.40 | 3.45 | 5.38 | 0100000 | s69817 | 0.0 | -0.083 | 0.60 | 0.65 |
| LONP2 | 7.69 | 7.06 | 8.51 | 7.51 | 8.50 | 1000001 | OAR14_16764006 | 5.2 | -0.083 | 0.83 | 0.88 |
| LRRC39 | 7.22 | 8.22 | 8.34 | 6.80 | 9.04 | 0100000 | OAR1_81920870 | 0.0 | 0.347 | 0.83 | 0.66 |
| LRRC6 | 5.59 | 6.69 | 7.83 | 6.53 | 7.84 | 0010000 | OAR9_22488751 | 0.0 | -0.111 | 0.83 | 0.89 |
| LTBP3 | 2.83 | 2.89 | 2.53 | 2.63 | 5.81 | 0001100 | s42358 | 0.0 | -0.111 | 0.54 | 0.60 |
| LYCAT | 8.01 | 7.50 | 8.32 | 7.85 | 8.83 | 0000001 | s22319 | 16.6 | -0.375 | 0.42 | 0.60 |
| MAML2 | 7.05 | 7.57 | 8.31 | 7.34 | 8.60 | 0000000 | s71238 | 0.0 | 0.458 | 0.88 | 0.65 |
| MAP3K5 | 7.12 | 5.50 | 6.55 | 7.49 | 7.17 | 0100010 | OAR8_66474331 | 0.0 | 0.208 | 0.77 | 0.67 |
| MAP4K5 | 6.77 | 7.08 | 7.66 | 7.01 | 6.02 | 0001000 | OAR7_44829954 | 0.0 | 0.139 | 0.73 | 0.66 |
| MAPK14 | 6.47 | 8.04 | 7.57 | 7.56 | 8.66 | 0001000 | OAR20_10297256 | 0.0 | 0.278 | 0.88 | 0.74 |
| MAPKAPK2 | 8.28 | 8.24 | 8.06 | 8.08 | 7.09 | 0001000 | s72402 | 0.0 | -0.028 | 0.83 | 0.85 |
| MARVELD1 | 2.03 | 2.93 | 4.16 | 3.30 | 2.88 | 0001100 | OAR22_21965564 | 6.2 | -0.111 | 0.65 | 0.70 |
| MARVELD2 | 5.63 | 4.84 | 4.71 | 5.65 | 6.39 | 0001110 | s30181 | 0.0 | 0.111 | 0.75 | 0.69 |
| MBOAT2 | 6.84 | 6.33 | 5.59 | 5.87 | 5.59 | 1000000 | OAR3_19854319 | 96.7 | -0.278 | 0.60 | 0.74 |
| MCM3AP | 7.73 | 5.01 | 7.29 | 6.80 | 7.24 | 1100001 | OAR1_285644761 | 0.0 | 0.014 | 0.54 | 0.53 |
| MDFIC | 6.46 | 7.71 | 8.23 | 7.04 | 8.23 | 0100000 | s23575 | 0.0 | -0.236 | 0.65 | 0.76 |
| MESDC2 | 3.51 | 3.53 | 3.65 | 4.75 | 4.33 | 0100000 | s34296 | 0.0 | -0.139 | 0.77 | 0.84 |
| METAP2 | 5.58 | 6.68 | 7.75 | 6.10 | 8.02 | 0100000 | OAR3_140347703 | 0.0 | -0.111 | 0.58 | 0.64 |
| MIER3 | 1.33 | 2.25 | 0.86 | 2.11 | 1.17 | 1000001 | OAR16_24428159 | 0.0 | 0.000 | 0.48 | 0.48 |
| MLLT4 | 4.79 | 5.16 | 4.88 | 5.48 | 4.41 | 0001001 | s17426 | 108.8 | 0.347 | 0.60 | 0.43 |
| MME | 5.45 | 5.46 | 7.26 | 6.08 | 6.70 | 1000000 | OAR1_248820310 | 127.2 | -0.278 | 0.56 | 0.70 |
| MOXD1 | 3.89 | 5.84 | 6.06 | 5.53 | 5.80 | 0000001 | OAR8_61599522 | 0.0 | -0.319 | 0.67 | 0.83 |
| MRPL3 | 7.15 | 6.54 | 6.81 | 6.91 | 7.83 | 0010000 | OAR1_277098838 | 29.7 | 0.347 | 0.85 | 0.68 |
| MRPL45 | 7.69 | 8.40 | 7.92 | 7.54 | 7.02 | 1000000 | OAR11_41010269 | 0.0 | 0.056 | 0.65 | 0.62 |
| MTM1 | 3.50 | 4.93 | 5.12 | 5.04 | 4.60 | 0001000 | s46003 | 0.0 | 0.125 | 0.60 | 0.54 |
| MUC12 | 5.05 | 5.74 | 6.48 | 6.29 | 6.85 | 0001000 | s20643 | 0.0 | -0.042 | 0.50 | 0.52 |
| MYH10 | 5.36 | 5.77 | 5.97 | 6.43 | 7.54 | 0001000 | s29973 | 1.0 | -0.569 | 0.46 | 0.74 |
| MYL1 | 11.97 | 10.94 | 11.37 | 11.43 | 11.29 | 1010110 | OAR2_223200146 | 0.0 | 0.208 | 0.81 | 0.71 |
| NEB | 5.90 | 5.10 | 6.78 | 7.05 | 6.43 | 1100000 | OAR2_166248181 | 0.0 | -0.083 | 0.50 | 0.54 |
| NPC1L1 | 3.63 | 4.73 | 5.75 | 4.10 | 4.59 | 0001000 | s38034 | 0.0 | 0.208 | 0.63 | 0.52 |
| NPEPL1 | 3.24 | 5.05 | 5.07 | 4.43 | 5.07 | 0000001 | OAR13_61989059 | 0.0 | -0.347 | 0.33 | 0.51 |
| NRK | 6.28 | 5.00 | 7.05 | 7.25 | 6.91 | 1100010 | OARX_81807292 | 36.8 | -0.264 | 0.60 | 0.74 |
| NRXN2 | 4.36 | 5.00 | 6.63 | 6.10 | 5.85 | 1000100 | s28165 | 44.1 | -0.292 | 0.58 | 0.73 |
| NSF | 4.52 | 4.87 | 5.29 | 5.89 | 6.76 | 0001000 | OAR11_47731535 | 0.0 | -0.153 | 0.81 | 0.89 |
| NTN1 | 6.15 | 6.87 | 7.79 | 7.04 | 6.72 | 0001000 | s48574 | 0.0 | -0.194 | 0.46 | 0.56 |
| NTRK3 | 5.61 | 6.82 | 7.93 | 6.25 | 7.21 | 0000100 | OAR18_18253258 | 0.0 | 0.250 | 0.58 | 0.46 |
| NUMB | 2.98 | 4.16 | 2.41 | 4.25 | 4.29 | 1001000 | OAR7_89063907 | 0.0 | 0.167 | 0.79 | 0.71 |
| NUP93 | 6.20 | 6.27 | 5.64 | 6.18 | 7.16 | 0001000 | s12233 | 0.0 | 0.181 | 0.67 | 0.58 |
| OCRL | 2.20 | 3.82 | 3.04 | 3.19 | 4.12 | 1001000 | OARX_112464882 | 0.0 | 0.264 | 0.67 | 0.53 |
| OGDH | 3.88 | 5.49 | 5.56 | 5.48 | 6.53 | 0001000 | s49251 | 0.0 | -0.028 | 0.75 | 0.76 |
| PACRG | 11.58 | 9.76 | 9.58 | 10.57 | 10.65 | 0010110 | OAR8_91790154 | 219.7 | -0.139 | 0.52 | 0.59 |
| PALLD | 3.64 | 5.79 | 5.21 | 4.78 | 5.44 | 0100010 | s23043 | 0.0 | 0.333 | 0.63 | 0.46 |
| PALM | 2.80 | 4.18 | 5.04 | 3.43 | 3.30 | 0001110 | s72060 | 0.0 | -0.333 | 0.40 | 0.56 |
| PDCD6IP | 11.76 | 10.65 | 10.73 | 11.16 | 11.04 | 0010000 | s20009 | 424.0 | -0.319 | 0.58 | 0.74 |
| PDE5A | 3.47 | 3.30 | 5.14 | 3.61 | 3.06 | 1001100 | OAR6_7822475 | 109.3 | -0.278 | 0.38 | 0.51 |
| PDGFRA | 6.12 | 5.33 | 6.22 | 5.99 | 6.10 | 0000000 | OAR6_76377079 | 116.0 | -0.611 | 0.31 | 0.62 |
| PDGFRL | 5.72 | 5.65 | 6.07 | 5.30 | 7.12 | 0001000 | s37569 | 0.0 | 0.000 | 0.88 | 0.88 |
| PDLIM5 | 9.72 | 8.24 | 8.03 | 9.07 | 9.07 | 0010110 | OAR6_33759226 | 63.4 | 0.194 | 0.92 | 0.82 |
| PEX5 | 8.67 | 7.28 | 6.95 | 8.33 | 8.89 | 0011110 | s03159 | 0.0 | -0.167 | 0.65 | 0.73 |
| PGM3 | 5.25 | 5.94 | 5.25 | 5.02 | 4.34 | 0001001 | OAR8_11440155 | 1.1 | 0.042 | 0.58 | 0.56 |
| PGM5 | 5.77 | 6.33 | 7.87 | 6.47 | 8.59 | 0001000 | DU485802_126 | 0.0 | 0.000 | 0.77 | 0.77 |
| PHLDB2 | 3.16 | 4.03 | 3.88 | 4.16 | 3.68 | 0000001 | OAR1_188675266 | 0.0 | 0.319 | 0.79 | 0.63 |
| PI4K2A | 4.01 | 5.43 | 5.24 | 5.11 | 5.23 | 1000001 | s53575 | 0.0 | -0.139 | 0.65 | 0.72 |
| PIAS4 | 5.97 | 6.48 | 6.73 | 6.83 | 6.23 | 0001000 | OAR5_20120209 | 0.0 | 0.375 | 0.71 | 0.52 |
| PLA2G12A | 5.61 | 7.28 | 6.76 | 6.87 | 8.21 | 1001000 | OAR6_18700834 | 0.0 | 0.000 | 0.65 | 0.65 |
| PLA2R1 | 7.70 | 7.29 | 7.71 | 7.53 | 8.89 | 0001000 | OAR2_157572121 | 0.0 | -0.236 | 0.63 | 0.74 |
| PLXNC1 | 5.10 | 8.07 | 7.78 | 7.50 | 7.99 | 0010111 | OAR3_139104149 | 3.3 | 0.319 | 0.81 | 0.65 |
| PM20D1 | 6.74 | 7.60 | 7.18 | 7.33 | 8.87 | 0001000 | s14484 | 0.0 | 0.236 | 0.71 | 0.59 |
| PMEPA1 | 6.28 | 8.20 | 7.77 | 7.36 | 8.31 | 1100000 | OAR13_62949442 | 0.0 | 0.056 | 0.69 | 0.66 |
| POLR2A | 5.77 | 4.96 | 6.51 | 6.20 | 6.19 | 1000000 | s36839 | 0.0 | -0.083 | 0.96 | 1.00 |
| PON1 | 2.00 | 4.57 | 3.09 | 4.72 | 4.12 | 1001001 | s31971 | 0.0 | -0.083 | 0.65 | 0.69 |
| PPP1R10 | 10.82 | 9.14 | 9.38 | 10.28 | 10.29 | 0010110 | OAR20_29921748 | 0.0 | 0.042 | 0.63 | 0.60 |
| PPP1R3A | 5.67 | 7.24 | 7.99 | 7.52 | 8.50 | 0010000 | OAR4_57697138 | 83.9 | 0.306 | 0.81 | 0.66 |
| PPP3CA | 3.81 | 4.37 | 5.90 | 4.45 | 4.37 | 0001100 | s53951 | 0.0 | -0.222 | 0.63 | 0.74 |
| PRLR | 2.68 | 4.87 | 5.69 | 4.36 | 4.23 | 0011110 | s15191 | 0.0 | -0.153 | 0.88 | 0.95 |
| PSAT1 | 9.16 | 9.27 | 8.85 | 8.61 | 9.21 | 0000000 | OAR2_62463225_X | 3.4 | 0.458 | 0.90 | 0.67 |
| PSMD14 | 8.25 | 7.47 | 7.90 | 8.01 | 8.42 | 0010001 | OAR2_156250273 | 0.0 | 0.222 | 0.65 | 0.53 |
| PTEN | 4.23 | 5.17 | 4.89 | 4.13 | 5.12 | 0100000 | OAR22_11189744 | 0.0 | -0.236 | 0.50 | 0.62 |
| PTGER3 | 5.57 | 6.70 | 6.95 | 6.41 | 5.47 | 0000001 | OAR1_48122363 | 0.0 | -0.236 | 0.54 | 0.66 |
| PTK2 | 5.06 | 6.12 | 5.75 | 4.78 | 5.73 | 0100000 | s22485 | 25.8 | 0.458 | 0.58 | 0.35 |
| PTPN18 | 6.99 | 7.19 | 7.87 | 7.54 | 8.69 | 0000000 | s23889 | 0.0 | 0.500 | 0.71 | 0.46 |
| PVRL1 | 6.72 | 8.48 | 8.37 | 7.44 | 9.11 | 0100000 | s27003 | 9.6 | 0.236 | 0.75 | 0.63 |
| RAB1A | 3.99 | 5.16 | 5.30 | 5.51 | 5.08 | 0000001 | OAR3_46103829 | 12.1 | 0.403 | 0.85 | 0.65 |
| RAB31 | 5.61 | 4.29 | 5.90 | 5.94 | 6.64 | 0100000 | s69800 | 0.0 | -0.389 | 0.50 | 0.69 |
| RAB7B | 5.05 | 5.05 | 6.50 | 5.18 | 5.01 | 0001100 | s06672 | 2.4 | -0.458 | 0.31 | 0.54 |
| RAD17 | 5.54 | 5.81 | 6.78 | 5.19 | 7.80 | 0000001 | OAR16_11208745 | 0.0 | 0.208 | 0.60 | 0.50 |
| RAD52 | 8.36 | 7.28 | 8.38 | 7.85 | 8.77 | 0000001 | OAR3_229469681_X | 0.0 | 0.111 | 0.56 | 0.51 |
| RALY | 4.17 | 4.44 | 5.20 | 4.47 | 6.74 | 0001000 | s12884 | 0.0 | 0.111 | 0.94 | 0.88 |
| RARRES1 | 4.68 | 6.19 | 5.23 | 5.40 | 7.12 | 0001000 | s02598 | 0.0 | -0.153 | 0.60 | 0.68 |
| RASGEF1B | 6.73 | 8.11 | 8.61 | 6.98 | 8.89 | 0100010 | s49765 | 406.4 | 0.264 | 0.88 | 0.74 |
| RBKS | 6.17 | 6.60 | 8.13 | 6.39 | 7.59 | 1000000 | OAR3_37136398 | 0.0 | -0.014 | 0.85 | 0.86 |
| RBM18 | 3.49 | 4.28 | 2.96 | 3.64 | 3.36 | 1000000 | OAR3_14634039_X | 0.0 | -0.319 | 0.33 | 0.49 |
| RBM7 | 10.15 | 9.04 | 9.16 | 9.62 | 9.80 | 0000100 | OAR15_25529117 | 0.0 | 0.097 | 0.94 | 0.89 |
| RDH12 | 6.10 | 8.03 | 8.40 | 7.48 | 9.11 | 0010010 | OAR7_83570505 | 1.2 | 0.111 | 0.54 | 0.49 |
| RHOBTB3 | 4.78 | 5.65 | 7.65 | 5.76 | 5.98 | 1000000 | OAR5_100623817 | 0.0 | -0.125 | 0.69 | 0.75 |
| RNF182 | 5.88 | 5.16 | 5.32 | 3.82 | 5.42 | 0100001 | OAR20_45364882 | 22.7 | 0.153 | 0.90 | 0.82 |
| RPUSD2 | 6.23 | 7.57 | 7.94 | 7.80 | 7.17 | 0000001 | OAR7_37386830 | 0.0 | -0.028 | 0.63 | 0.64 |
| S100A9 | 2.53 | 4.99 | 4.47 | 4.44 | 4.77 | 0000001 | s33937 | 1.4 | 0.028 | 0.73 | 0.72 |
| SARG | 4.97 | 6.95 | 6.07 | 6.39 | 6.14 | 1000001 | OAR12_1565631 | 0.0 | 0.000 | 0.73 | 0.73 |
| SEC11A | 3.09 | 2.67 | 3.84 | 4.32 | 3.66 | 0100000 | OAR18_22035684 | 0.0 | 0.250 | 0.63 | 0.50 |
| SECISBP2 | 4.37 | 5.01 | 4.28 | 4.40 | 4.73 | 1100000 | OAR2_24404686 | 0.0 | 0.042 | 0.90 | 0.88 |
| SETMAR | 9.97 | 9.86 | 9.65 | 9.51 | 9.92 | 0000000 | OAR19_23119299 | 73.1 | -0.486 | 0.35 | 0.60 |
| SFRP4 | 5.76 | 6.14 | 7.70 | 6.53 | 5.95 | 0001000 | OAR4_52576142 | 0.0 | -0.222 | 0.75 | 0.86 |
| SGPL1 | 7.26 | 5.57 | 5.05 | 6.16 | 6.28 | 0000100 | OAR25_28070242 | 1.2 | -0.083 | 0.48 | 0.52 |
| SH3D19 | 2.84 | 3.26 | 3.82 | 4.26 | 3.39 | 0001000 | OAR17_7125292 | 24.4 | 0.417 | 0.85 | 0.65 |
| SHISA9 | 7.23 | 7.57 | 8.31 | 8.38 | 8.88 | 0000000 | OAR24_13240468 | 0.0 | 0.542 | 0.90 | 0.63 |
| SILV | 8.16 | 8.87 | 8.51 | 9.15 | 9.22 | 0000001 | s59363 | 0.0 | -0.153 | 0.79 | 0.87 |
| SIN3A | 10.28 | 8.54 | 9.31 | 9.95 | 9.96 | 0100010 | OAR18_33752500 | 0.0 | 0.153 | 1.00 | 0.92 |
| SLAIN2 | 3.63 | 5.62 | 5.37 | 4.72 | 6.30 | 0100000 | OAR6_73460055 | 8.1 | 0.125 | 0.67 | 0.60 |
| SLC11A2 | 7.76 | 7.44 | 7.52 | 7.89 | 8.05 | 0000000 | OAR3_144283427 | 11.6 | 0.472 | 0.88 | 0.64 |
| SLC1A3 | 10.09 | 8.81 | 8.89 | 9.73 | 9.78 | 0010110 | s34284 | 17.3 | 0.153 | 0.71 | 0.63 |
| SLC2A1 | 5.20 | 6.47 | 6.19 | 5.59 | 6.11 | 0000001 | s60080 | 1.4 | 0.139 | 0.90 | 0.83 |
| SLC2A3 | 1.90 | 3.92 | 4.19 | 3.89 | 3.08 | 0000001 | s63713 | 1.2 | 0.083 | 0.94 | 0.90 |
| SLC39A8 | 3.29 | 3.72 | 4.02 | 4.94 | 4.78 | 0001000 | OAR6_25861437_X | 0.0 | 0.319 | 0.73 | 0.57 |
| SLC4A2 | 4.71 | 7.04 | 7.46 | 6.99 | 7.56 | 0001001 | OAR4_120564652 | 0.6 | -0.083 | 0.56 | 0.60 |
| SLC7A5 | 5.88 | 4.99 | 5.55 | 6.63 | 5.60 | 0000000 | s55065 | 0.0 | -0.486 | 0.40 | 0.64 |
| SLC7A8 | 3.66 | 4.64 | 3.66 | 5.98 | 4.95 | 1000001 | OAR7_22252106 | 0.1 | -0.042 | 0.69 | 0.71 |
| SMARCD2 | 6.25 | 7.23 | 7.88 | 6.22 | 6.10 | 0001000 | s46223 | 0.0 | 0.069 | 0.63 | 0.59 |
| SMYD3 | 7.11 | 8.25 | 8.22 | 7.54 | 8.78 | 0000000 | OAR12_32749330 | 0.0 | -0.486 | 0.50 | 0.74 |
| SNX4 | 3.69 | 5.64 | 4.66 | 5.25 | 5.05 | 0000001 | OAR1_202894635 | 1.4 | 0.056 | 0.44 | 0.41 |
| SORBS2 | 5.77 | 7.15 | 7.28 | 7.31 | 8.37 | 0001000 | OAR26_17518698 | 0.0 | 0.111 | 0.73 | 0.67 |
| SP3 | 6.20 | 7.36 | 8.33 | 7.49 | 8.29 | 0000000 | OAR2_143746835 | 194.9 | -0.472 | 0.54 | 0.78 |
| SPAST | 6.60 | 8.53 | 8.31 | 7.24 | 8.86 | 0100000 | OAR3_97385996 | 0.0 | 0.139 | 0.69 | 0.62 |
| SPINK5 | 9.58 | 9.19 | 8.29 | 9.54 | 9.67 | 1011100 | OAR5_61924626 | 0.0 | 0.042 | 0.79 | 0.77 |
| SPP1 | 6.93 | 6.88 | 7.51 | 7.65 | 8.63 | 0001000 | OAR6_40855809 | 0.0 | 0.111 | 0.81 | 0.76 |
| ST3GAL3 | 4.83 | 8.01 | 7.78 | 5.72 | 7.30 | 1100011 | s74275 | 0.0 | -0.167 | 0.52 | 0.60 |
| STARD9 | 3.98 | 5.26 | 5.53 | 5.36 | 5.68 | 0011110 | OAR7_39225246 | 0.0 | -0.208 | 0.52 | 0.63 |
| STAT1 | 6.68 | 8.66 | 8.44 | 6.98 | 8.65 | 0100010 | OAR2_203559364 | 0.0 | -0.333 | 0.40 | 0.56 |
| SUGT1 | 7.25 | 5.35 | 6.94 | 6.89 | 7.20 | 1000000 | s53147 | 0.0 | 0.222 | 0.69 | 0.58 |
| SUPT3H | 8.19 | 7.00 | 8.33 | 7.96 | 8.49 | 1100011 | OAR20_19253173 | 15.6 | 0.181 | 0.83 | 0.74 |
| SUV39H2 | 6.96 | 8.30 | 8.03 | 8.30 | 8.94 | 0000001 | OAR13_31489025 | 0.0 | -0.167 | 0.44 | 0.52 |
| SYVN1 | 10.98 | 9.29 | 8.89 | 9.80 | 10.10 | 0010110 | s72465 | 0.0 | -0.014 | 0.60 | 0.61 |
| TAF7 | 3.54 | 4.01 | 3.05 | 3.91 | 4.22 | 1001001 | OAR5_54040603 | 1.0 | 0.056 | 0.58 | 0.56 |
| TBC1D2 | 5.17 | 5.04 | 7.20 | 5.91 | 6.85 | 0101000 | OAR2_52697563 | 0.7 | 0.139 | 0.90 | 0.83 |
| TBC1D8 | 3.47 | 3.93 | 5.32 | 5.14 | 5.62 | 0001110 | s29822 | 0.0 | 0.278 | 0.69 | 0.55 |
| TBX15 | 4.38 | 3.67 | 4.96 | 5.29 | 4.53 | 1100000 | OAR1_101890858 | 0.0 | -0.125 | 0.54 | 0.60 |
| TGFBR2 | 9.81 | 8.34 | 7.75 | 9.34 | 9.08 | 1010110 | s62323 | 9.3 | -0.181 | 0.56 | 0.65 |
| TGFBR3 | 6.08 | 5.53 | 6.46 | 5.96 | 5.65 | 0001000 | s66349 | 0.0 | 0.139 | 0.75 | 0.68 |
| TGIF2 | 7.14 | 7.35 | 7.29 | 7.36 | 8.09 | 1000000 | s63108 | 0.0 | -0.125 | 0.79 | 0.85 |
| THBS2 | 5.52 | 3.84 | 6.39 | 6.03 | 4.91 | 1101000 | s07249 | 38.7 | -0.194 | 0.56 | 0.66 |
| TIPARP | 4.06 | 5.36 | 5.17 | 4.03 | 4.84 | 0100010 | OAR1_246909447 | 0.0 | 0.125 | 0.92 | 0.85 |
| TM9SF4 | 5.70 | 5.31 | 5.48 | 5.48 | 5.10 | 0001000 | s22254 | 0.0 | -0.264 | 0.50 | 0.63 |
| TMEM158 | 6.18 | 6.95 | 6.76 | 6.41 | 7.77 | 0000000 | s04445 | 56.1 | 0.764 | 0.79 | 0.41 |
| TMEM45A | 7.86 | 6.87 | 7.77 | 8.08 | 7.15 | 0001000 | OAR1_176418046 | 2.7 | -0.083 | 0.63 | 0.67 |
| TMEM87B | 5.88 | 7.97 | 7.87 | 7.06 | 7.31 | 0000010 | s09051 | 0.8 | 0.083 | 0.83 | 0.79 |
| TNC | 8.28 | 8.20 | 8.52 | 8.19 | 7.60 | 0001000 | s64483 | 49.7 | 0.375 | 0.83 | 0.65 |
| TNFAIP8 | 4.12 | 4.92 | 4.13 | 3.58 | 4.20 | 1100000 | OAR5_35442371 | 0.0 | 0.236 | 0.83 | 0.72 |
| TNRC6B | 5.23 | 4.37 | 6.40 | 4.67 | 6.53 | 0000001 | OAR3_233491871 | 0.0 | 0.194 | 0.56 | 0.47 |
| TOPORS | 5.93 | 7.39 | 8.21 | 7.62 | 8.32 | 0010100 | OAR2_108072651 | 0.0 | -0.028 | 0.77 | 0.78 |
| TPD52 | 6.85 | 5.08 | 6.02 | 7.05 | 7.43 | 0100000 | OAR9_58845489 | 0.0 | 0.250 | 0.73 | 0.60 |
| TPD52L1 | 4.43 | 2.79 | 4.91 | 6.01 | 4.76 | 0100010 | s74581 | 0.0 | 0.153 | 0.77 | 0.69 |
| TPK1 | 1.38 | 3.42 | 2.84 | 2.05 | 4.50 | 1101000 | OAR4_115148402 | 305.6 | 0.264 | 0.88 | 0.74 |
| TRAPPC2L | 5.87 | 7.06 | 7.83 | 6.87 | 7.81 | 0010110 | s60777 | 0.0 | -0.111 | 0.90 | 0.95 |
| TRIM27 | 5.36 | 5.05 | 6.59 | 6.25 | 5.84 | 1001100 | s71648 | 0.0 | 0.097 | 0.54 | 0.49 |
| TRIM29 | 1.28 | 2.48 | 2.73 | 3.33 | 1.71 | 0001101 | OAR15_31730166 | 0.0 | 0.153 | 0.90 | 0.82 |
| TRPM8 | 5.32 | 7.40 | 7.04 | 6.26 | 8.43 | 1001000 | OAR1_6373388 | 26.7 | -0.222 | 0.63 | 0.74 |
| TRPS1 | 3.16 | 4.70 | 4.97 | 4.48 | 4.46 | 0000110 | OAR9_64654880 | 17.6 | 0.250 | 0.96 | 0.83 |
| UBA6 | 1.93 | 4.09 | 3.56 | 2.94 | 3.39 | 0100010 | OAR6_91038014 | 0.0 | 0.111 | 0.81 | 0.76 |
| UBAC2 | 7.28 | 7.29 | 8.47 | 6.93 | 8.64 | 0000001 | s58237 | 0.0 | -0.125 | 0.73 | 0.79 |
| UBE2S | 4.00 | 5.08 | 4.34 | 4.15 | 4.86 | 1100000 | s67978 | 0.0 | 0.083 | 0.65 | 0.60 |
| UBN1 | 4.03 | 5.44 | 5.03 | 5.81 | 4.34 | 0001001 | OAR24_4666768 | 0.0 | -0.139 | 0.63 | 0.69 |
| UGCGL1 | 4.83 | 5.41 | 5.21 | 5.70 | 6.76 | 0001000 | s42677 | 0.0 | 0.139 | 0.92 | 0.85 |
| ULK2 | 6.17 | 8.07 | 7.83 | 7.08 | 8.30 | 0100010 | s48054 | 0.0 | -0.042 | 0.77 | 0.79 |
| UNC50 | 4.36 | 4.87 | 6.04 | 5.35 | 7.05 | 0001000 | OAR3_108930523 | 1.8 | 0.222 | 0.75 | 0.64 |
| UQCRFS1 | 4.37 | 4.87 | 6.12 | 5.02 | 6.18 | 0000000 | OAR14_614123 | 145.1 | -0.556 | 0.31 | 0.59 |
| USP12 | 7.63 | 7.92 | 8.69 | 8.08 | 7.44 | 1000000 | OAR10_33168014 | 0.0 | 0.083 | 0.83 | 0.79 |
| USP7 | 5.64 | 6.21 | 7.07 | 6.42 | 7.96 | 0001000 | s50307 | 0.0 | 0.139 | 0.92 | 0.85 |
| VDAC1 | 7.57 | 6.42 | 8.03 | 8.22 | 8.49 | 1100000 | s57816 | 2.2 | 0.000 | 1.00 | 1.00 |
| VGLL4 | 3.63 | 3.97 | 5.10 | 5.40 | 5.00 | 1100100 | OAR19_58842437 | 0.0 | -0.028 | 0.56 | 0.58 |
| VPS28 | 8.20 | 8.20 | 8.39 | 8.34 | 7.39 | 0001000 | s41927 | 0.0 | -0.208 | 0.56 | 0.67 |
| WBSCR17 | 3.43 | 4.73 | 5.47 | 5.01 | 5.12 | 0000001 | s64838 | 54.7 | -0.167 | 0.48 | 0.56 |
| WDFY2 | 13.16 | 11.69 | 11.35 | 11.59 | 12.65 | 0010100 | s03832 | 0.0 | 0.417 | 0.75 | 0.54 |
| WDR70 | 7.54 | 5.08 | 5.96 | 7.36 | 6.44 | 0110010 | OAR16_40016364 | 0.0 | -0.333 | 0.50 | 0.67 |
| XDH | 5.64 | 7.11 | 7.87 | 6.92 | 8.52 | 0001000 | OAR3_97895496 | 0.0 | 0.236 | 0.54 | 0.42 |
| YEATS2 | 5.52 | 5.35 | 5.56 | 4.95 | 4.67 | 0001000 | OAR1_217543314 | 0.0 | -0.111 | 0.63 | 0.68 |
| YIPF4 | 9.72 | 9.08 | 9.25 | 9.55 | 9.74 | 0010000 | OAR3_97206388 | 0.0 | -0.139 | 0.50 | 0.57 |
| YIPF5 | 4.69 | 4.15 | 3.86 | 4.70 | 4.78 | 0000110 | OAR5_57024851 | 62.9 | 0.264 | 0.67 | 0.53 |
| YPEL2 | 4.65 | 4.94 | 4.58 | 5.53 | 5.62 | 0001000 | s15694 | 0.0 | -0.222 | 0.67 | 0.78 |
| ZBTB10 | 6.61 | 6.62 | 8.34 | 7.89 | 8.85 | 1000000 | s60973 | 171.4 | -0.361 | 0.58 | 0.76 |
| ZC3HAV1 | 6.00 | 8.20 | 8.30 | 7.33 | 8.65 | 0000000 | OAR4_109083033 | 5.8 | -0.472 | 0.56 | 0.80 |
| ZFR | 4.84 | 5.87 | 7.31 | 7.21 | 6.36 | 1011100 | OAR16_44759148 | 26.7 | -0.111 | 0.90 | 0.95 |
| ZNF187 | 5.07 | 5.83 | 5.46 | 5.27 | 5.89 | 1000000 | OAR20_32327104 | 0.0 | -0.153 | 0.90 | 0.97 |
| ZNF367 | 5.29 | 7.81 | 7.40 | 6.03 | 6.90 | 1000001 | OAR2_30690903 | 0.0 | -0.069 | 0.75 | 0.78 |
| ZNF608 | 7.04 | 5.22 | 7.31 | 7.42 | 6.37 | 1100000 | s62606 | 89.7 | -0.278 | 0.54 | 0.68 |
|  |  |  |  |  |  |  |  |  |  |  |  |
|  |  |  |  |  |  |  |  |  |  |  |  |
